# Gaucher disease protects against tuberculosis

**DOI:** 10.1073/pnas.2217673120

**Published:** 2023-02-06

**Authors:** Jingwen Fan, Victoria L. Hale, Lindsey T. Lelieveld, Laura J. Whitworth, Elisabeth M. Busch-Nentwich, Mark Troll, Paul H. Edelstein, Timothy M. Cox, Francisco J. Roca, Johannes M. F. G. Aerts, Lalita Ramakrishnan

**Affiliations:** ^a^Molecular Immunity Unit, Cambridge Institute of Therapeutic Immunology and Infectious Diseases, Department of Medicine, University of Cambridge, Cambridge CB2 0QH, UK; ^b^MRC Laboratory of Molecular Biology, Cambridge CB2 0QH, UK; ^c^Department of Medical Biochemistry, Leiden Institute of Chemistry, Leiden University 2333 CC, Leiden, The Netherlands; ^d^School of Biological and Behavioral Sciences, Queen Mary University of London, London E1 4NS, UK; ^e^Department of Pathology and Laboratory Medicine, Perelman School of Medicine, University of Pennsylvania, Philadelphia PA 19104; ^f^Department of Medicine, University of Cambridge, Cambridge CB2 0QQ, UK; ^g^Department of Biochemistry and Molecular Biology B and Immunology, University of Murcia, Murcia 30120, Spain; ^h^Biomedical Research Institute of Murcia Pascual Parrilla (IMIB-Arrixaca), Murcia 30120, Spain

**Keywords:** Gaucher disease, tuberculosis resistance, lysosomal glucosylsphingosine, macrophages, zebrafish

## Abstract

Biallelic mutations in the glucocerebrosidase (*GBA1*) gene cause Gaucher disease, characterized by lysosomal accumulation of glucosylceramide and glucosylsphingosine in macrophages. Gaucher and other lysosomal diseases occur with high frequency in Ashkenazi Jews. It has been proposed that the underlying mutations confer a selective advantage, in particular conferring protection against tuberculosis. Here, using a zebrafish Gaucher disease model, we find that the mutation *GBA1* N370S, predominant among Ashkenazi Jews, increases resistance to tuberculosis through the microbicidal activity of glucosylsphingosine in macrophage lysosomes. Consistent with lysosomal accumulation occurring only in homozygotes, heterozygotes remain susceptible to tuberculosis. Thus, our findings reveal a mechanistic basis for protection against tuberculosis by *GBA1* N370S and provide biological plausibility for its selection if the relatively mild deleterious effects in homozygotes were offset by significant protection against tuberculosis, a rampant killer of the young in Europe through the Middle Ages into the 19th century.

## Significance

Gaucher disease is a recessively inherited disorder in which the lipids glucosylceramide and glucosylsphingosine accumulate in lysosomes of macrophages. Macrophages are the first immune cells to engulf infecting bacteria, and we find that glucosylsphingosine increases their ability to kill *Mycobacterium tuberculosis* that causes tuberculosis*.*Gaucher disease due to a particular mutation is frequent in Ashkenazi Jews. Since from the middle ages they were often confined to areas of high tuberculosis prevalence, it has been proposed that the mutation prevailed because heterozygotes, who do not accumulate lipids or manifest Gaucher disease, were protected. Our findings raise the possibility that selection operated on homozygotes manifesting mild forms of Gaucher disease who were protected against tuberculosis which would often have been fatal.

Tuberculosis (TB) features multiple interactions of *Mycobacterium tuberculosis* (Mtb) with host macrophages, each with the potential to determine if the infection will progress or be cleared (1). Zebrafish develop TB-like disease when infected with their natural pathogen *Mycobacterium marinum* (Mm), a close relative of Mtb (1). In particular, the optically transparent and genetically and pharmacologically tractable zebrafish larva has enabled delineation of the early steps of TB pathogenesis and the host–*Mycobacterium* interactions that shape them, with zebrafish findings providing insights into human TB pathogenesis and treatment and forming the basis for preclinical and clinical studies and clinical trials (1–14).

Through a zebrafish genetic screen, we previously identified a mutant with a lysosomal storage disorder due to a deficiency in lysosomal cysteine cathepsins that was hypersusceptible to mycobacterial infection (7). We found that lysosomal storage in macrophages causes hypersusceptibility by impairing their migration into the developing tuberculous granuloma, thus causing pathological necrosis which promotes mycobacterial growth (7). In contrast to the ultrarare cathepsin deficiencies causing accumulation of proteinaceous material, lysosomal diseases that impair recycling of lipids are far more prevalent (15). Accordingly, we sought to determine whether a lysosomal disease associated with pathological storage of lipids affected susceptibility to TB. Glucocerebrosidase (GBA) deficiency, which causes Gaucher disease, is one of the most common lysosomal disorders (15, 16) and is of particular interest because it principally affects macrophages—host cells that interact early and critically with mycobacteria. In Gaucher disease, the lysosomal compartment of macrophages expands and becomes engorged with sphingolipid to assume a storage phenotype (17, 18). Moreover, these cells, widely known as Gaucher cells, have been shown to have defective migration (17, 18).

The zebrafish is an ideal model in which to address the question of how the macrophage lysosomal storage of Gaucher disease might impact TB; over the last few years, it has come into its own as a facile model for Gaucher disease that recapitulates its sphingolipid accumulation and key multisystem pathological manifestations—hematopoietic, including the hallmark macrophage lysosomal storage, visceral, bony and skeletal, and neuronopathic (19–23). The use of activity-based probes and mass spectrometric techniques has enabled the detailed biochemical assays to confirm in the zebrafish Gaucher disease model the metabolic shifts seen in human Gaucher disease (21). Importantly, the use of the zebrafish model has clarified the role of the two glucocerebrosidases GBA1 (lysosomal facing) and GBA2 (cytosolic facing) and the downstream enzyme, lysosomal acid ceramidase, in lipid accumulation and pathogenesis (21, 24).

Based on our findings with the cathepsin mutant, we predicted that GBA-deficient zebrafish would be hypersusceptible to mycobacterial infection. Unexpectedly, GBA-deficient zebrafish larvae were resistant to both Mm and Mtb, despite the fish having cardinal manifestations of human Gaucher disease, particularly overt macrophage lysosomal storage and accompanying migration defects. We have delineated the resistance mechanism and shown it to be operant from very early in infection and relevant in the context of the common Ashkenazi Jewish N370S Gaucher disease allele (25–28). Our findings shed light on the decades-long debate about the persistence of this allele—selection versus founder effect resulting in genetic drift (29–33). We provide biological evidence in support of its TB-driven selection over the centuries.

## Results

### *gba1* Mutant Zebrafish Develop Human Gaucher Disease Manifestations.

**Fig. 1.**
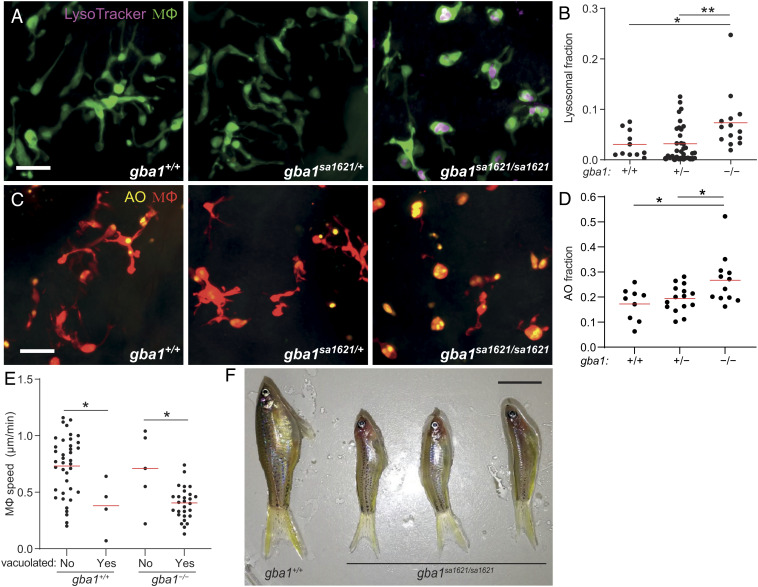
*gba1* mutant zebrafish manifest Gaucher disease. (*A*) Maximum intensity projection of pseudocolored representative confocal images of YFP-expressing macrophages stained with LysoTracker Red in 3 dpf zebrafish brains. (Scale bar, 20 μm.) (*B*) LysoTracker Red volume per macrophage in 3 dpf brains. Each point represents the mean volume fraction per macrophage in each brain. Horizontal red bars, means; *P < 0.05; **P < 0.01 (one-way ANOVA with Tukey’s posttest). Representative of at least three independent experiments. (*C*) Maximum intensity projection of representative confocal images of tdTomato-expressing macrophages stained with AO in 3 dpf zebrafish brains. (Scale bar, 20 μm.) (*D*) AO volume per macrophage in the brains of 3 dpf zebrafish. Each point represents the average AO volume fraction per macrophage in each fish. Horizontal red bars, means; *P < 0.05 (one-way ANOVA with Tukey’s posttest). (*E*) Homeostatic migration speed of normal and vacuolated macrophages in the brains of 3 dpf zebrafish. Each point represents the mean speed of an individual macrophage from the same animal per indicated genotype during 2 h observation. Horizontal red bars, means. *P < 0.05 (one-way ANOVA with Tukey’s posttest). Representative of two to three animals for each genotype. (*F*) Representative images of three *gba1^sa1621/sa1621^* fish and their wild-type sibling at 77 d after fertilization (dpf). (Scale bar, 1 cm.)

We examined a zebrafish mutant in the orthologous gene *gba1* (*gba1^sa1621^*) with a premature stop mutation in the region encoding the GBA catalytic domain (*SI Appendix*, Fig. S1 *A* and *B*). Like the cathepsin-deficient zebrafish (7), at 3 d after fertilization (dpf), *gba1^sa1621/sa1621^* mutants had an increased proportion of enlarged, rounded, brain-resident macrophages (microglia) with LysoTracker staining showing enlarged lysosomes containing accumulated cell debris as evidenced by acridine orange (AO) staining (Fig. 1 *A*–*D*). This macrophage phenotype is similar to that described for human Gaucher disease, and like human *GBA1* heterozygotes, *gba1^sa1621^* heterozygotes had normal macrophages (17, 18) (Fig. 1 *A*–*D*). Macrophages manifesting lysosomal storage moved slowly, as expected (7) (Fig. 1*E*). *gba1^sa1621^* homozygotes grew into early adulthood but were smaller in size and had curved spines that were previously reported for other zebrafish *gba1* mutants, recapitulating the growth retardation and kyphosis seen in many Gaucher disease patients (19, 20, 24, 34) (Fig. 1*F*). Moreover, between 75 and 80 d of age, all exhibited abnormal swimming characterized by a spinning motion (Movie S1) (19). This indicates involvement of the nervous system, which also complicates severe human Gaucher disease (34). None of the wild-type or heterozygous siblings manifested any of these pathological phenotypes during the 2- to 2.5-y observation period.

### *gba1* Mutant Zebrafish Are Resistant to Mm Infection.

**Fig. 2.**
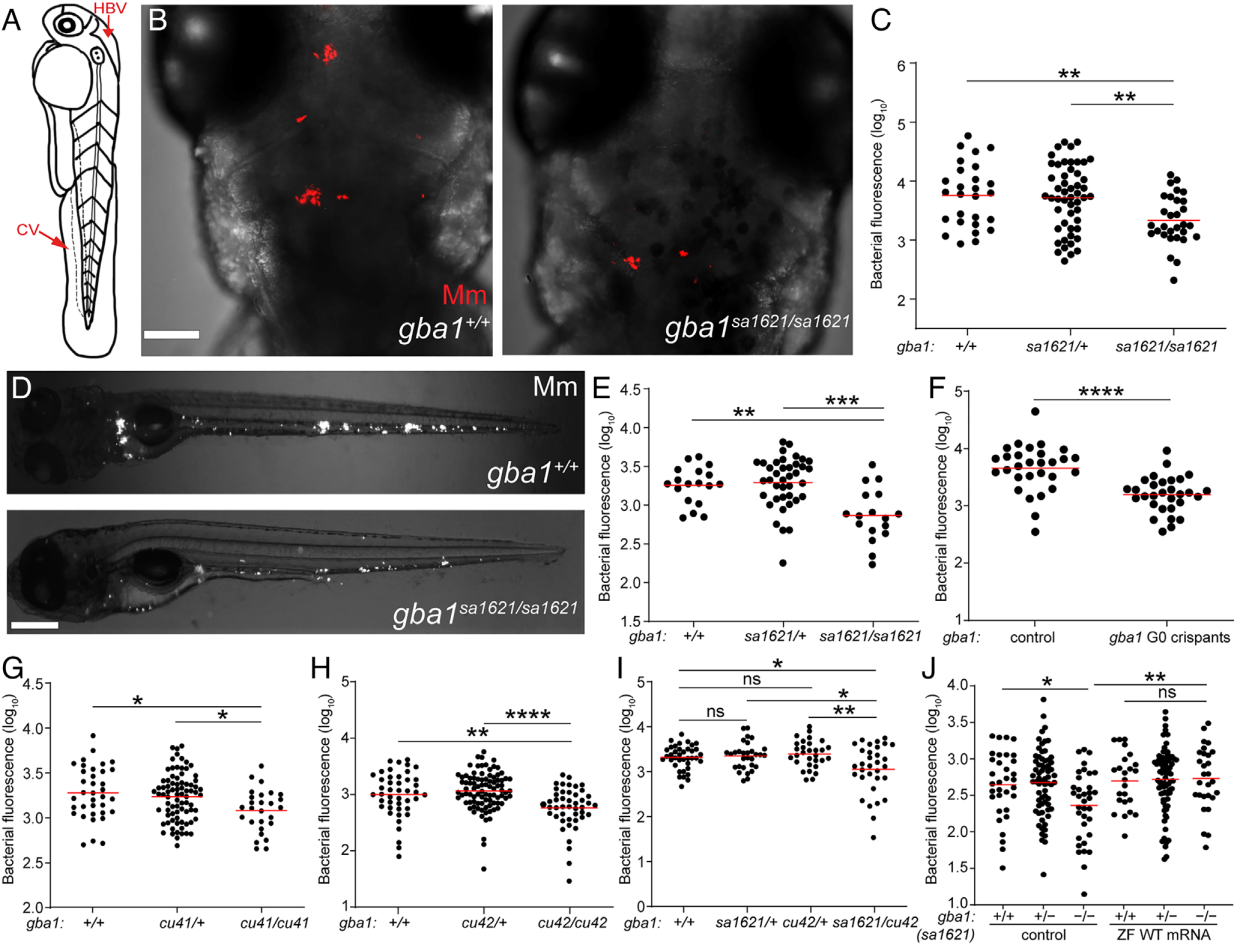
*gba1* mutant zebrafish are resistant to Mm infection. (*A*) Illustration of a zebrafish larva showing the HBV and CV injection sites. (*B*) Maximum intensity projection of representative confocal images of zebrafish larval HBV at 3 dpi after HBV infection with 100 to 150 Mm. (Scale bar, 80 μm.) (*C*) Quantification of HBV bacterial burden measured by fluorescence per animal from (*B*). Horizontal red bars, means; **P < 0.01 (one-way ANOVA with Tukey’s posttest). Representative of more than three independent experiments. (*D*) Representative images of zebrafish larvae at 5 dpi after CV infection with 200 to 300 Mm. (Scale bar, 300 μm.) (*E*) Quantification of the bacterial burden measured by fluorescence per animal from (*D*). Horizontal bars, means; **P < 0.01; ***P < 0.001 (one-way ANOVA with Tukey’s posttest). Representative of more than three independent experiments. (*F*) Bacterial burden measured by fluorescence per animal in wild-type and *gba1* G0 crispants at 5 dpi after CV infection with 200 to 300 Mm. Horizontal bars, means; ****P < 0.0001 (Student’s unpaired *t* test). Representative of more than three independent experiments. (*G*) Bacterial burden measured by fluorescence in 5 dpi *gba1^cu41/+^* incross larvae after CV infection with 200 to 300 Mm. Horizontal bars, means; *P < 0.05 (one-way ANOVA with Tukey’s posttest). Representative of two independent experiments. (*H*) Bacterial burden measured by fluorescence in 5 dpi *gba1^cu42/+^* incross larvae after CV infection with 200 to 300 Mm. Horizontal bars, means; **P < 0.01; ****P < 0.0001 (one-way ANOVA with Tukey’s posttest). Representative of more than three independent experiments. (*I*) Bacterial burden measured by fluorescence in wild-type, *sa1621* and *cu42* heterozygotes, and *sa1621/cu42* compound mutant siblings at 5 dpi after CV infection with 200 to 300 Mm. Horizontal bars, means; ns, not significant; *P < 0.05; **P < 0.01 (one-way ANOVA with Tukey’s posttest). (*J*) Bacterial burden measured by fluorescence in *gba1^sa1621/+^* incross larvae, injected with zebrafish WT *gba1* mRNA (200 ng/μL) or vehicle control, 5 dpi after CV infection with 200 to 300 Mm. Horizontal bars, means; ns, not significant; *P < 0.05; **P < 0.01 (one-way ANOVA with Tukey’s posttest). Representative of two independent experiments.

We next tested the susceptibility of *gba1* mutants to mycobacteria. An antisense *gba1* morpholino had resembled the cathepsin-deficient mutant, displaying macrophage lysosomal storage and increased susceptibility, similar to the cathepsin-deficient mutants (7). Because morpholinos may have off-target and/or toxic effects (35), it was important to test the *gba1* mutant fish for susceptibility. We infected 2 dpf animals from a *gba1^sa1621^* heterozygote incross with fluorescent Mm in the hindbrain ventricle (HBV), an epithelium-lined cavity where mycobacteria interact initially with first-responding resident macrophages before monocyte–*Mycobacterium* interactions become dominant as granulomas form (36) (Fig. 2*A*). Contrary to expectation, we found that they were Mm resistant. Fewer bacteria were present in the HBV at 3 d after infection (dpi) in the homozygous *gba1* mutant fish than in their wild-type siblings (Fig. 2 *B* and *C*). Heterozygotes had wild-type bacterial burdens (Fig. 2*C*). Intravenous injection of bacteria into the caudal vein (CV) where mycobacteria interact directly with monocytes (36) (Fig. 2*A*) also showed the same resistance phenotype (Fig. 2 *D* and *E*). To ensure that the resistance phenotype was specifically due to the *gba1* mutation, we created *gba1* G0 crispants using three different guide RNAs (*SI Appendix*, Fig. S1*A*). The pooled *gba1* G0 crispants were also resistant to Mm infection (Fig. 2*F*). We then generated two individual mutants *gba1^cu41^* and *gba1^cu42^* (*SI Appendix*, Fig. S1 *A* and *B*). Like the *gba1^sa1621^* mutants, both mutants were small with curved spines and developed abnormal swimming by about 80 d. Likewise, *gba1^cu41^* and *gba1^cu42^* homozygotes were resistant to Mm infection (Fig. 2 *G* and *H*). Compound *sa1621*/*cu42* heterozygotes were also resistant (Fig. 2*I*), and restoration of GBA using zebrafish *gba1* RNA eliminated resistance of *gba1* mutants (Fig. 2*J*), confirming that the *gba1* mutation caused resistance.

We confirmed that the previously-observed increased susceptibility of the *gba1* morphants (7) was indeed due to morpholino toxicity (35). Injection of the *gba1* morpholino (7) into a *gba1^sa1621^* heterozygote incross revealed high toxicity of this morpholino: most of the morphants had a profound abnormal development, with distorted bodies and aberrant circulation regardless of their *gba1* mutant status (*SI Appendix*, Fig. S2*A*). Their lysosomal storage phenotype was more profound than that of the mutants alone—likely due to exacerbated cell death resulting from morpholino toxicity during development—and the morpholino abolished the resistance of the mutants (*SI Appendix*, Fig. S2 *B* and *C*). Moreover, as before, we observed in the morphants, regardless of mutant status, bacterial cording, a phenotype associated with macrophage death (7) (*SI Appendix*, Fig. S2*D*). We therefore concluded that while the lysosomal storage phenotype of the morphants was due to GBA deficiency, the other phenotypes were due to toxicity and/or off-target effects which were affecting macrophage viability (35). The true phenotype of GBA deficiency is macrophage lysosomal storage coupled with macrophage resistance and not susceptibility.

### GBA-Deficient Macrophages Have Increased Mycobactericidal Capacity.

**Fig. 3.**
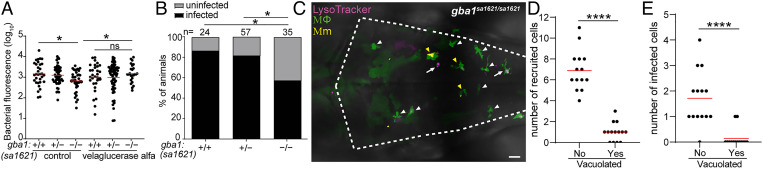
*gba1* mutant macrophages are bactericidal. (*A*) Bacterial burden measured by fluorescence in 3 dpi *gba1^sa1621/+^* incross larvae treated with 100 units/mL velaglucerase alfa or vehicle control after HBV infection with 100 to 150 Mm. Horizontal bars, means; ns, not significant; *P < 0.05 (one-way ANOVA with Tukey’s posttest). Representative of two independent experiments. (*B*) Percentage of infected larvae at 5 dpi after infection of the HBV with a single Mm bacterium. *P < 0.05 (Fisher’s exact test). Representative of two independent experiments. (*C*) Maximum intensity projection of pseudocolored representative confocal image of YFP-expressing macrophages stained with LysoTracker Red in the brain of 2 dpf *gba1* mutant larvae at 6 h after infection (hpi) in the HBV. Arrows mark vacuolated macrophages which have not phagocytosed Mm. White arrowheads mark nonvacuolated macrophages which have not phagocytosed Mm. Yellow arrowheads mark nonvacuolated macrophages which have phagocytosed Mm. (Scale bar, 20 μm.) (*D*) Quantification of nonvacuolated and vacuolated macrophages that are recruited to HBV in *gba1* mutant larvae from (*C*). Each point represents the total number of recruited nonvacuolated or vacuolated macrophages in each fish. Horizontal red bars, means. ****P < 0.0001 (Student’s unpaired *t* test). (*E*) Quantification of nonvacuolated and vacuolated macrophages with phagocytosed Mm from (*C*). Each point represents the total number of infected nonvacuolated or vacuolated macrophages in each fish. Horizontal red bars, means. ****P < 0.0001 (Student’s unpaired *t* test).

How could the migratory defect of *gba1* mutant macrophages be reconciled with resistance rather than susceptibility to Mm? We reasoned that resistance must manifest at the earliest stage of infection before the shortfall of recruited macrophages that leads to granuloma breakdown from macrophage necrosis. If so, then GBA-deficient macrophages must be better able to restrict growth of the infecting mycobacteria, enabling rapid reduction of the infection burden even before the granuloma stage. To test whether this was the case, we used velaglucerase alfa, a mannose-terminated human GBA protein that is taken up into macrophages and an effective enzyme replacement therapy for patients with Gaucher disease (37). Administration of velaglucerase alfa eliminated mutant resistance, suggesting that GBA-deficient macrophages have increased intrinsic ability to restrict mycobacterial growth (Fig. 3*A*).

To determine if this reflected an increased ability to kill mycobacteria, i.e., increased microbicidal capacity, we performed an “infectivity assay” where we infected zebrafish in the HBV with a single Mm (36). Because mycobacteria are rapidly phagocytosed by macrophages, the frequency of animals with infection progression versus clearance at 5 dpi is a reliable indicator of macrophage microbicidal capacity (36) (*SI Appendix*, Fig. S3). Significantly more *gba1* mutant zebrafish had cleared infection, confirming that their macrophages were more microbicidal to mycobacteria (Fig. 3*B*). Thus, GBA-deficient brain-resident macrophages possess increased microbicidal capacity that causes increased killing of phagocytosed Mm.

Since we had observed the resistance phenotype after CV infection where the mycobacteria are phagocytosed by blood monocytes of the caudal hematopoietic tissue (CHT) (Fig. 2 *D* and *E*), these blood monocytes must also be more microbicidal. However, the CHT monocytes did not manifest overt lysosomal storage (*SI Appendix*, Fig. S4). [We attribute the more apparent storage in brain macrophages to increased substrate accumulation as they are constantly engulfing apoptotic neurons during brain development at this stage (38).] This finding suggested that GBA-deficient macrophages and monocytes have increased microbicidal capacity even if they have not developed the overt storage phenotype of the pathological Gaucher cell (17, 18).

This model would be consistent with our prior findings that macrophages with advanced lysosomal storage cannot migrate to mycobacteria and participate in the infection (7). We had already shown that in the *gba1* mutant, the vacuolated macrophages with obvious lysosomal storage had a homeostatic migration defect (Fig. 1*E*). To confirm whether they also had defective migration to infecting mycobacteria, we infected Mm into the HBV, which first recruits proximate brain-resident macrophages (36). Whereas most macrophages in *gba1* mutant brains had the Gaucher cell phenotype (Fig. 1*A*), these were a distinct minority among those that were recruited to infection (Fig. 3 *C* and *D*). Rather, infection almost exclusively recruited the few macrophages that were phenotypically normal (Fig. 3 *C* and *D*); those with the Gaucher cell phenotype were found predominantly in the midbrain away from the HBV infection site. Consequently, these cells were the ones most likely to be infected (Fig. 3 *C* and *E*). Thus, in GBA-deficient animals, both tissue-resident macrophages and monocytes have enhanced mycobactericidal capacity even if they do not manifest an obvious lysosomal storage phenotype.

### Glucosylsphingosine, Which Accumulates in GBA-Deficient Macrophage Lysosomes, Is Mycobactericidal In Vitro.

**Fig. 4.**
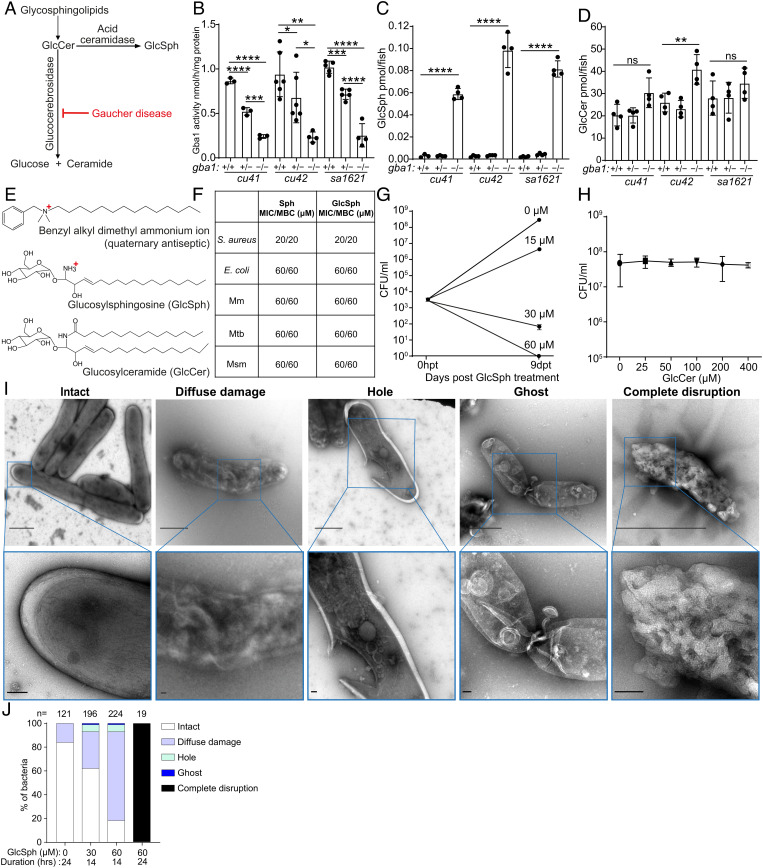
Glucosylsphingosine is bactericidal in vitro. (*A*) Representation of the pathway leading to glucosylceramide and glucosylsphingosine accumulation in Gaucher disease. GlcCer, glucosylceramide. GlcSph, glucosylsphingosine. (*B*) Glucocerebrosidase enzyme activity (nmol/mg) in 5 dpf *gba1 cu41, cu42,* and *sa1621* mutants and their wild-type siblings. Mean ± SD; *P < 0.05; **P < 0.01; ***P < 0.001; ****P < 0.0001 (one-way ANOVA with Tukey’s posttest). (*C*) GlcSph (pmol/fish) in 5 dpf *gba1 cu41, cu42,* and *sa1621* mutants and their wild-type siblings. Mean ± S.D; ****P < 0.0001 (one-way ANOVA with Tukey’s posttest). Representative of two independent experiments. (*D*) GlcCer (pmol/fish) in 5 dpf *gba1 cu41, cu42,* and *sa1621* mutants and their wild-type siblings. Mean ± SD; ns, not significant; **P < 0.01 (one-way ANOVA with Tukey’s posttest). Representative of two independent experiments. (*E*) Chemical structures of benzyl alkyl dimethyl ammonium ion, GlcSph, and GlcCer. (*F*) MIC/MBC table of *Staphylococcus aureus*, *Escherichia coli,* Mm, Mtb, and *M. smegmatis* treated with Sph and GlcSph. Representative of one to two independent experiments performed in duplicate. (*G*) Mm killing by GlcSph. Mean CFU/mL; vertical bars, upper and lower values of the two technical replicates. (*H*) Mean Mm (CFU/mL) after incubation with increasing GlcCer concentrations for 9 d. Vertical bars, upper and lower values of the two technical replicates (starting concentration, 2.8 × 10^3^ CFU/mL). Representative of two independent experiments. (*I*) Negative stain TEM images of Msm treated with GlcSph, representative of the types of damage seen. [Scale bars, 1 μm (*Top*) and 100 nm (*Bottom*).] (*J*) Quantification of the types of damage seen in (*H*) for various GlcSph concentrations.

One explanation for the increased mycobactericidal capacity of GBA-deficient macrophages was that their accumulated lysosomal products could be directly microbicidal to mycobacteria. We first tested whether the accumulated lysosomal products could account for the increased microbicidal capacity of GBA-deficient macrophages. GBA deficiency causes the accumulation of glucosylceramide which is converted to glucosylsphingosine by lysosomal acid ceramidase (39–41) (Fig. 4*A*). Glucosylsphingosine is consistently elevated in Gaucher disease macrophage lysosomes, and its increased concentration in blood is used as a diagnostic biomarker (39, 42). We confirmed that at 5 dpf, all three zebrafish mutants had reduced GBA activity and increased concentrations of glucosylsphingosine, as expected from a similar analysis of another zebrafish *gba1* null mutant (21) (Fig. 4 *B* and *C*). Also, as in the previously published mutant, neither glucosylceramide nor other sphingolipid concentrations were invariably elevated in these mutants (21) (Fig. 4*D* and *SI Appendix*, Fig. S5). The partial reduction of enzymatic activity in the heterozygotes did not result in any glucosylsphingosine accumulation as in the published zebrafish mutant and in human heterozygous carriers (21, 39) (Fig. 4 *B* and *C*).

Free sphingosines are broadly microbicidal to gram-positive and gram-negative bacteria in vitro and are thought to contribute to the antimicrobial activity of normal skin (43). This is expected as they are cationic surfactants similar to antiseptics such as the quaternary ammonium compounds, long known to be bactericidal; this is because the positively charged groups are strongly attracted to negatively charged groups in bacterial membranes, where they impair fluidity, cause leakage, and ultimately disrupt the membranes (44, 45) (Fig. 4*E*). Consistent with its predicted bactericidal activity, sphingosine had the same minimum inhibitory concentration (MIC) and minimum bactericidal concentration (MBC) against the gram-positive bacterium *Staphylococcus aureus* and the gram-negative bacterium *Escherichia coli* (46) (Fig. 4*F*). Consistent with the membrane-disrupting activity of cationic surfactants, sphingosine was broadly bactericidal to all three mycobacterial species tested, Mm, Mtb, and *Mycobacterium smegmatis* (Msm), a rapidly growing environmental nonpathogenic species (Fig. 4*F*). The lower glucosylsphingosine MIC for *Staphylococcus aureus* (20 μM) than for *Escherichia coli* and mycobacteria (60 μM) may be because the latter bacteria have a multilayer membrane, which could increase resistance to its membrane-disrupting action (47). Importantly, glucosylsphingosine had the same bactericidal activity as sphingosine to all the bacteria tested (Fig. 4*F*). At concentrations lower than its MBC, glucosylsphingosine was still growth inhibitory to Mm, slightly inhibiting and completely inhibiting growth at 15 and 30 μM concentrations, respectively (Fig. 4*G*). Moreover, as predicted because it is a neutral lipid, glucosylceramide did not inhibit bacterial growth even at the highest concentration tested, 400 μM (Fig. 4 *E* and *H*).

Consistent with its membrane-disrupting action, sphingosine has been shown to produce disruption of the *Staphylococcus aureus* cell wall (43). We confirmed by negative stain transmission electron microscopy (TEM) that glucosylsphingosine likewise caused ultrastructural damage to *Staphylococcus aureus* membranes (*SI Appendix*, Fig. S6). Using Msm, we then showed that glucosylsphingosine caused similar damage to mycobacterial membranes in a concentration- and time-dependent manner (Fig. 4 *I* and *J*). Thus, glucosylsphingosine, but not glucosylceramide, is bactericidal to mycobacteria in vitro through its membrane-disrupting activity. This finding is consistent with the membrane-disrupting mycobactericidal activity of glucosylsphingosine being responsible for the increased macrophage microbicidal capacity of GBA-deficient macrophages we had observed in vivo.

### Accumulated Lysosomal Glucosylsphingosine Is Responsible for Enhanced Mycobacterial Killing by GBA-Deficient Macrophages In Vivo.

**Fig. 5.**
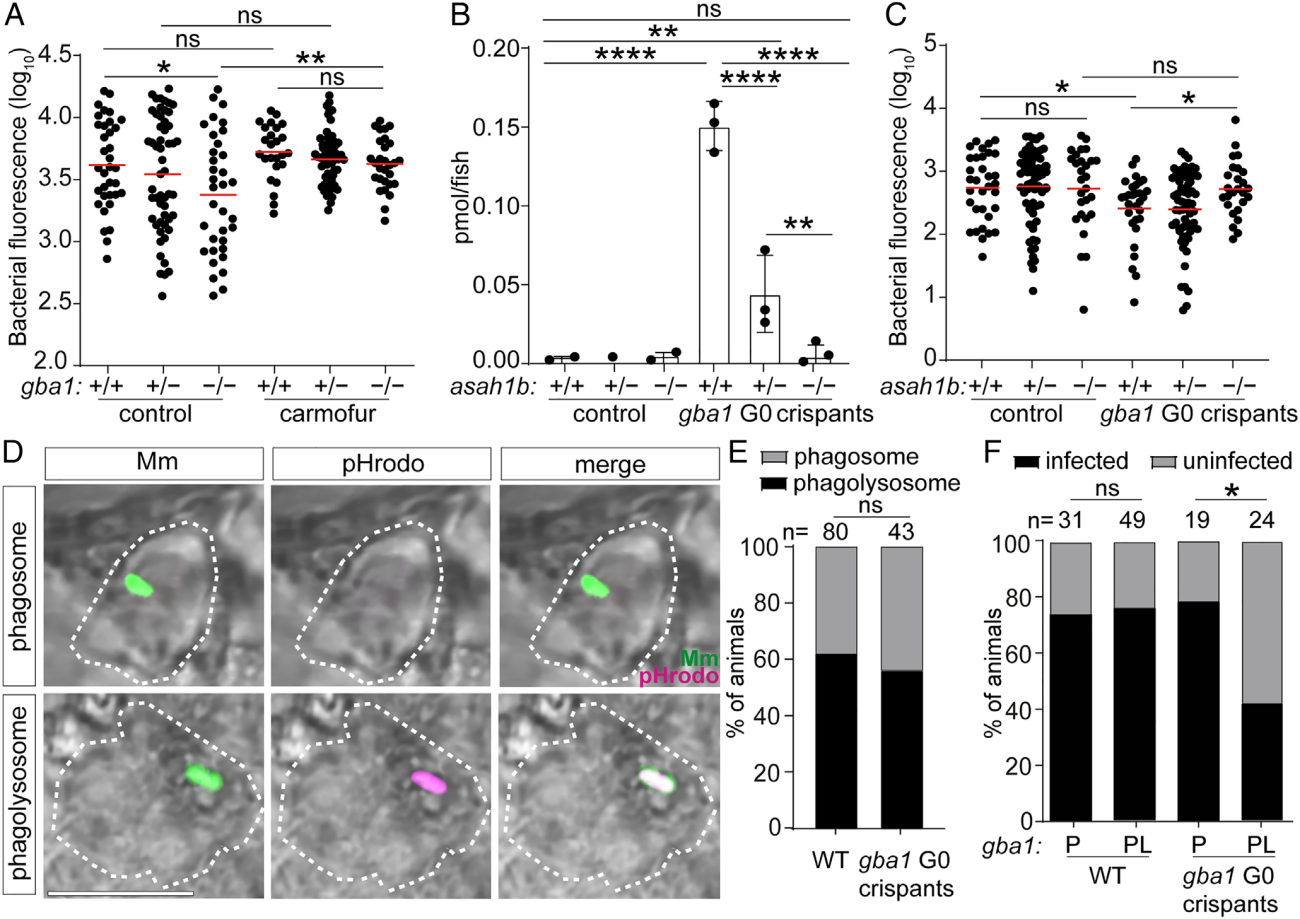
Glucosylsphingosine is bactericidal in vivo. (*A*) Bacterial burden measured by fluorescence in *gba1^sa1621/+^* incross larvae treated with 0.5 μM carmofur or vehicle control 5 dpi after CV infection with 200 to 300 Mm. Horizontal bars, means; ns, not significant; *P < 0.05; **P < 0.01 (one-way ANOVA with Tukey’s posttest). Representative of two independent experiments. (*B*) GlcSph (pmol/fish) in *asah1b* incross larvae that are either wild type or G0 crispant for *gba1*. Mean ± SD; ns, not significant; **P < 0.01; ****P < 0.0001 (one-way ANOVA with Tukey’s posttest). Representative of two independent experiments. (*C*) Bacterial burden measured by fluorescence in *asah1b* incross larvae that are either wild type or G0 crispant for *gba1* 5 dpi after CV infection with 200 to 300 Mm. Horizontal bars, means; ns, not significant; *P < 0.05 (one-way ANOVA with Tukey’s posttest). (*D*) Representative confocal images showing phagosome-localized Mm (green) and phagolysosome-localized Mm (green plus magenta) inside zebrafish macrophages (bright field, dashed lines) at 12 hpi. (Scale bar, 10 μm.) (*E*) Percentage of animals in which the single infecting Mm bacterium was localized to a phagosome or a phagolysosome; ns, not significant (Fisher’s exact test). Representative of two to three independent experiments. (*F*) Percentage of infected wild-type and *gba1* G0 crispants at 5 dpi after HBV infection with a single Mm bacterium. P, phagosome; PL, phagolysosome; ns, not significant; *P < 0.05 (Fisher’s exact test). Representative of two to three independent experiments.

Alternatively, or additionally, resistance in vivo could be an indirect consequence of GBA deficiency, attributable to induction of microbicidal lysosomal enzymes (e.g., lysozyme) and cytokines (e.g., tumor necrosis factor) characteristic of Gaucher disease (1, 15, 48). Therefore, we performed experiments to determine if the accumulated glucosylsphingosine was necessary and sufficient for the increased macrophage microbicidal capacity of GBA-deficient macrophages in vivo. If so, then resistance of *gba1* mutants should be abolished by blocking conversion of the accumulating glucosylceramide to glucosylsphingosine by inhibition of acid ceramidase (Fig. 4*A*). Pharmacological inhibition of acid ceramidase with carmofur (49) eliminated *gba1* mutant resistance (Fig. 5*A*). Next, we superimposed a genetic mutation in acid ceramidase (zebrafish *asah1b*) on *gba1* mutants by creating *gba1* G0 crispants in a cross of *asah1b* heterozygotes. The *gba1–asahb1* double mutants did not accumulate glucosylsphingosine, as shown before (Fig. 5*B*) (24). Importantly, they lost resistance to Mm (Fig. 5*C*). The *asah1b* heterozygotes had residual accumulation of glucosylsphingosine, and this was sufficient to confer resistance (Fig. 5 *B* and *C*). Thus, the increased resistance of *gba1* mutant animals is specifically due to the accumulated glucosylsphingosine, which is necessary for resistance in vivo.

To determine whether the accumulated glucosylsphingosine in macrophages was sufficient for resistance in vivo, we took advantage of an important feature of the intramacrophage lifestyle of mycobacteria, namely that upon infecting macrophages, Mm and Mtb phagosomes frequently but not always fuse with lysosomes (50). Therefore, if glucosylsphingosine is sufficient for increased macrophage mycobactericidal activity, then only the mycobacteria in the fused phagolysosome compartments should be killed more in GBA-deficient macrophages as compared to wild-type macrophages. If, on the other hand, increased inflammation in GBA-deficient macrophages additionally contributes to the increased killing, then mycobacteria in unfused phagosomes should also have a greater likelihood of being killed. By infecting zebrafish larvae with green fluorescent bacteria that have been labeled with pHrodo, a dye that fluoresces red in acidified compartments, we were able to distinguish bacteria in fused phagosome–lysosome compartments (green plus red fluorescence) from those in unfused phagosomes (only green fluorescence) (50) (Fig. 5*D* and *SI Appendix*, Fig. S7). The fate of these individual bacteria—growth versus clearance—can be determined using the infectivity assay at 5 dpi (50) (*SI Appendix*, Fig. S7). We found, as expected, that approximately 60% of the bacteria were found in fused phagolysosomes in wild-type animals (50); this proportion was not altered in *gba1* mutants (Fig. 5*E*). Also as predicted on account of the acid tolerance of mycobacteria, in wild-type animals, phagosome–lysosome fusion did not increase clearance of infection (50) (Fig. 5*F*). By contrast, in *gba1* mutants, bacteria in fused phagosome–lysosomes were significantly more likely to be killed (Fig. 5*F*). However, there was no increased killing of bacteria in the unfused phagosomes of GBA-deficient macrophages (Fig. 5*F*). Thus, the increased bacterial clearance in *gba1* mutants was entirely due to increased microbicidal capacity specifically of their lysosomes (Fig. 5*F*). Therefore, the glucosylsphingosine that accumulates in GBA-deficient macrophage lysosomes is both necessary and sufficient for the increased mycobacterial killing observed in vivo at the early stages of infection, which leads to increased clearing of infection. Indeed, our results show that the subcellular location where bacterial killing occurs is precisely where the formation of glucosylsphingosine is maximum. These findings rule out a role for the increased inflammation associated with Gaucher disease in early resistance and enhanced bacterial clearing.

### Common N370S GBA Mutation Confers Resistance to TB.

**Fig. 6.**
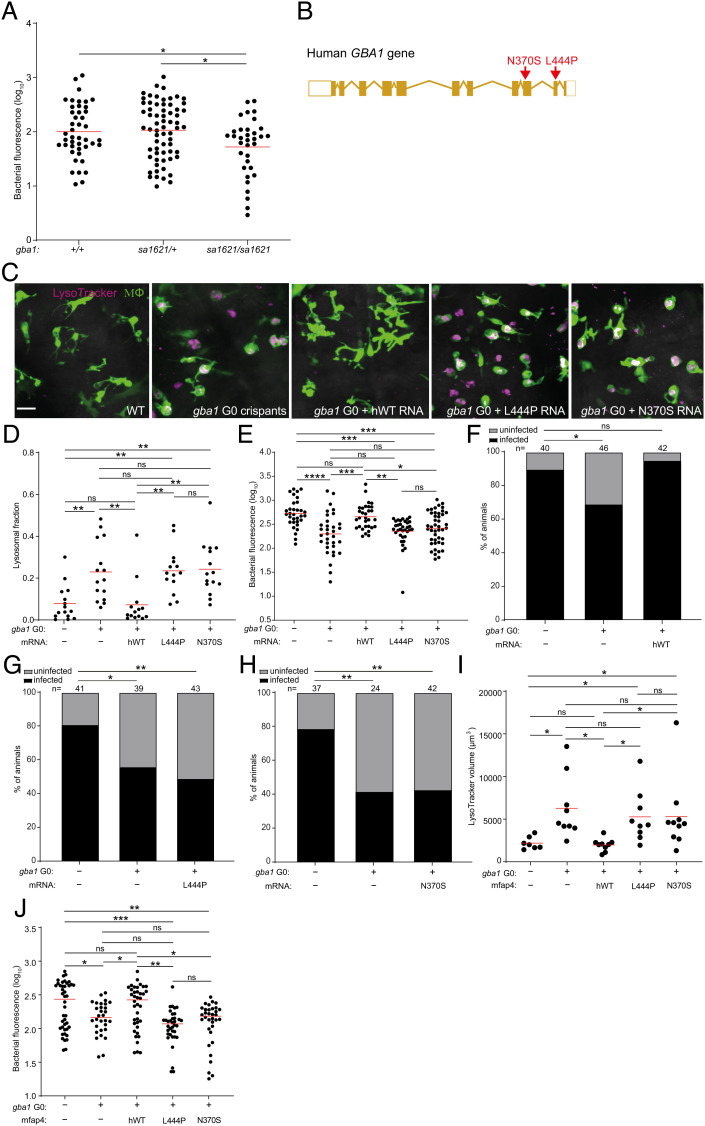
Common human *GBA1* mutants confer resistance to TB. (*A*) Bacterial burden measured by fluorescence in *gba1^sa1621/+^* incross larvae 5 dpi after CV infection of 400 to 500 Mtb. Horizontal bars, means. *P < 0.05 (one-way ANOVA with Tukey’s posttest). (*B*) Schematic diagram showing the location of the two human *GBA1* mutations studied. (*C*) Maximum intensity projection of representative pseudocolored confocal images of brain macrophages (yellow fluorescent) stained with LysoTracker Red in wild-type larvae, *gba1* G0 crispants, and *gba1* G0 crispants expressing human wild-type, L444P or N370S mutant *GBA1* mRNA. (Scale bar, 20 μm.) (*D*) Mean LysoTracker Red volume per macrophage in 3 dpf animals from (*C*). Each point represents mean LysoTracker Red volume fraction per macrophage in each fish. Horizontal bars, means; ns, not significant; **P < 0.01 (one-way ANOVA with Tukey’s posttest). Representative of two to three independent experiments. (*E*) Bacterial burden measured by fluorescence in 5 dpi wild-type, *gba1* G0 crispants and *gba1* G0 crispants expressing human wild-type, L444P or N370S mutant *GBA1* mRNA after CV infection with 600 to 700 Mtb. Horizontal bars, means; ns, not significant; *P < 0.05; **P < 0.01; ***P < 0.001; ****P < 0.0001 (one-way ANOVA with Tukey’s posttest). Representative of two independent experiments. (*F*) Percentage of infected wild-type animals, *gba1* G0 crispants and *gba1* G0 crispants expressing human wild-type *GBA1* mRNA fish at 5 dpi after HBV infection with a single Mtb bacterium. ns, not significant; *P < 0.05 (Fisher’s exact test). Representative of two independent experiments. (*G*) Percentage of infected wild-type animals, *gba1* G0 crispants and *gba1* G0 crispants expressing human L444P mutant *GBA1* mRNA at 5 dpi after HBV infection with a single Mtb bacterium. *P < 0.05 and **P < 0.01 (Fisher’s exact test). (*H*) Percentage of infected wild-type animals, *gba1* G0 crispants and *gba1* G0 crispants expressing human N370S mutant *GBA1* mRNA at 5 dpi after HBV infection with a single Mtb bacterium. **P < 0.01 (Fisher’s exact test). Representative of two independent experiments. (*I*) Total LysoTracker Red volume in 3 dpf wild-type, *gba1* G0 crispants and *gba1* G0 crispants expressing macrophage-specific human wild-type, L444P or N370S mutant *GBA1* gene. Each point represents total LysoTracker Red volume in each fish. Horizontal bars, means. ns, not significant and *P < 0.05 (one-way ANOVA with Tukey’s posttest). (*J*) Bacterial burden measured by fluorescence in 5 dpi wild-type, *gba1* G0 crispants and *gba1* G0 crispants expressing macrophage-specific human wild-type, L444P or N370S mutant *GBA1* gene after CV infection with 600 to 700 Mtb. Horizontal bars, means; ns, not significant; *P < 0.05; **P < 0.01; ***P < 0.001 (one-way ANOVA with Tukey’s posttest).

Our cumulative findings led to the question of whether mutant alleles associated with Gaucher disease protect humans against TB. This question is pertinent to the debate on why the disease incidence persists at high frequency in the Ashkenazi Jewish population (1/800 births versus 1/40,000-60,000 births in other populations) (17). Previous hypotheses of a link between Gaucher disease and protection against TB in Ashkenazi Jews have proposed a model of heterozygote advantage wherein TB resistance in heterozygotes might offset the deleterious effects of the disease in homozygotes (29, 32). However, consistent with glucosylsphingosine accumulation occurring only in homozygotes, we had found that only homozygotes, not heterozygotes, were resistant to Mm (Fig. 2 *C*, *E*, and *G*–*I*). Following up on our finding that glucosylsphingosine also kills Mtb in vitro (Fig. 4*F*), we showed that *gba1* mutant homozygotes were also resistant to Mtb (Fig. 6*A*). Again, heterozygotes remained susceptible (Fig. 6*A*). Thus, our findings support a link between Gaucher disease allele homozygosity and protection against TB directly through the microbicidal activity of accumulated glucosylsphingosine in macrophage lysosomes rather than the proposed model of heterozygote advantage presumably through some indirect means.

The proposal that homozygosity for a disease-causing allele could be positively selected presents a conundrum. This conundrum may be resolved by the widely variable features of disease in patients homozygous for *GBA1* N370S (c.1226 A > G; also referred to as p.Asn409Ser—N409S—to include a signal peptide) (Fig. 6*B*). More than 300 mutations of *GBA1* have been described worldwide (16, 26), but the evidence for selective advantage is centered around the N370S allele, which occurs at high frequency in the Ashkenazi Jewish population, with between one in 18 and one in 11 persons a carrier of the mutation (25–28). Persons homozygous for GBA N370S typically manifest the mildest (nonneuronopathic, Type 1) form of Gaucher disease with age of onset 10 to 30 y later than for other Gaucher genotypes (25, 51). Indeed, half to two-thirds of individuals homozygous for GBA N370S remain asymptomatic, and fertility is normal even among homozygotes with Gaucher disease–associated pathologies (16, 25, 52). These characteristics of N370S Gaucher disease may be consistent with a model where protection of homozygotes against TB offsets the deleterious effect of the mutation on the Ashkenazi population as a whole.

For the homozygote protection model for GBA N370S to be plausible, two essential conditions must be met: 1) glucosylsphingosine must accumulate at mycobactericidal concentrations in GBA N370S homozygote macrophages as in GBA-deficient zebrafish macrophages, and 2) zebrafish expressing only the human GBA N370S mutation must be resistant to Mtb similar to zebrafish GBA null mutants. To see whether the first condition was met, we calculated glucosylsphingosine concentrations in macrophages from published reports of amounts determined in macrophages prepared from humans homozygous for the GBA N370S mutation and from healthy human macrophages treated with a glucocerebrosidase inhibitor; each is reported to be ≈0.6 nmol/mg protein (18, 39) (*SI Appendix*, Box S1). We calculated these intramacrophage concentrations to be ≈200 μM (*SI Appendix*, Box S1). This is in excellent agreement with glucosylsphingosine concentrations of 19 μM (95% CI 9 to 29) in whole spleen tissue obtained from N370S compound heterozygotes with Type 1 nonneuronopathic Gaucher disease (*SI Appendix*, Box S1), in which the lipid-laden macrophages or Gaucher cells accumulate but still contribute only a minor fraction of tissue mass (18, 39). Thus, the intramacrophage concentration of glucosylsphingosine exceeds its MIC for Mtb (60 μM), and as a base, the molecule will be further concentrated in the acidic lysosome compartment of macrophages as positively charged micelles with potentiated bactericidal activity (53, 54).

To test the second condition that must be met, namely whether the human GBA N370S mutation results in resistance to Mtb, we turned to genetic complementation of the zebrafish *gba1* G0 crispants with human *GBA* RNAs, which have been used previously to rescue bone abnormalities in *gba1* mutant zebrafish (20). Wild-type human *GBA* RNA should reverse their macrophage lysosomal storage phenotype and eliminate Mtb resistance [as shown with zebrafish *gba1* RNA in Mm (Fig. 2*J*)]. In contrast, neither of the two phenotypes should be altered by RNA bearing the L444P mutation (c.1448 T > C and also referred to as p.Leu483Pro, L483P), a severe Gaucher disease allele with minimal residual glucocerebrosidase activity in vitro (26, 55). As predicted, injection of wild-type *GBA* RNA, but not L444P *GBA* RNA, rescued lysosomal storage and abolished Mtb resistance, demonstrating the validity of this complementation assay (Fig. 6 *C*–*E*). We next tested whether the zebrafish *gba1* mutant phenotypes would be reversed by the human N370S GBA allele. Despite its greater residual glucocerebrosidase activity (10 to 15% of wild type) (56), N370S *GBA* RNA also failed to rescue lysosomal storage and abolish Mtb resistance (Fig. 6 *C*–*E*). To test whether the N370S GBA mutation renders Mtb-infected macrophages microbicidal, we performed the infectivity assay. Wild-type *GBA* RNA restored the reduced infectivity of the *gba1* G0 crispants to wild-type levels, whereas neither mutant RNA did (Fig. 6 *F*–*H*).

The experiments above used transient, ubiquitously expressed human RNAs to evaluate human GBA alleles for protection against TB. In an independent approach, we created stable transgenic zebrafish lines expressing either wild type or each of the two mutant GBA alleles selectively in macrophages (*SI Appendix*, Fig. S8). GBA1 crispants were created either in wild type or in each of the three transgenic backgrounds, resulting, respectively, in GBA1 null mutants or mutants expressing human wild type, L444P or N370S GBA1, but only in their macrophages. As was the case with the transiently expressed human RNAs, macrophage-specific expression of human wild-type GBA1 reversed both the macrophage lysosomal phenotype and the resistance to TB of the GBA null mutants, whereas neither the L444P nor the N370S GBA alleles did (Fig. 6 *I* and *J*). We could not test the infectivity phenotypes in this set of experiments because of the paucity of available transgenic mutants—5 to 10-fold more animals are required to obtain sufficient animals that received single bacteria; however, since this is a macrophage-intrinsic phenotype, there is no reason for the results to be different from those obtained for the animals complemented with the ubiquitously expressed RNAs. Thus, in the zebrafish, the human N370S GBA allele has the same effect as more severe Gaucher disease mutations, resulting in lysosomal storage and conferring increased resistance to TB through increased macrophage microbicidal activity against Mtb.

## Discussion

Our findings provide support for the long-standing hypothesis that Gaucher disease, including that caused by the common Ashkenazi Jewish N370S mutation, confers resistance to TB by enhancing macrophage mycobactericidal activity. In keeping with biallelic inheritance where only homozygotes and not heterozygotes accumulate the culpable substrate, we find that only homozygotes and not heterozygotes are resistant to TB. Importantly, we find that in Gaucher disease, macrophage mycobactericidal activity is enhanced from the earliest step of infection when macrophages first encounter mycobacteria. In the context of the Mtb life cycle, this would occur when alveolar macrophages in the lung phagocytose the bacteria, increasing early clearance of infection (1). Enhanced macrophage mycobactericidal capacity would also increase the chances of clearing infection in subsequent steps of infection, i.e., in the granuloma. We recognize that our findings raise an apparent paradox: for macrophages to be recruited to and become infected with mycobacteria, they must be motile, which is unlikely the case for the classic Gaucher cell with substantial lysosomal storage (7, 18). However, we find that even macrophages without overt lysosomal storage have increased microbicidal capacity, leading us to infer that the macrophages in Gaucher disease that mediate resistance have accumulated smaller amounts of glucosylsphingosine, enough to restrict mycobacterial growth, although not enough to induce the Gaucher cell phenotype. In support of this, while alveolar macrophages can manifest lysosomal storage in Gaucher disease, frank Gaucher cells within airspaces are a rare occurrence in patients with type 1 disease—the nonneuronopathic phenotype of N370S homozygotes (57–59). Our estimate that even 15 μM concentrations of glucosylsphingosine—far lower than the ~200 μM concentrations found in GBA-deficient macrophages—inhibit mycobacterial growth lends further support to this argument. Macrophages with this much lower glucosylsphingosine concentration may well lack the classical Gaucher cell phenotype but possess enhanced ability to restrict mycobacteria, particularly given the concentration of the compound in the form of positively charged micelles in mycobacterial phagolysosomes, which we have demonstrated to be mycobactericidal in GBA-deficient zebrafish.

Our zebrafish findings are supported by a very recent report showing that cultured iPSC-derived macrophages from a patient with Gaucher disease (homozygous for the L444P allele) were microbicidal to Mtb, and this resistance was abolished upon Crispr correction to wild type (60). Our work delineating the molecular and cellular basis of this microbicidal activity highlights the advantages of the zebrafish with its amenability to genetic and pharmacological manipulations and the unique live subcellular imaging possible in this transparent model organism. Particularly germane to this study, GBA-deficient macrophages will have different lipid loads at different times, depending on when they have ingested dying cells (7). The use of the zebrafish has allowed us to study GBA-deficient macrophages, including those carrying the human N370S mutation in a true in vivo context, while in the process of carrying out their dual homeostatic scavenger and antimicrobial functions.

Our findings have potentially important genetic implications. They offer biological evidence related to the question of whether GBA N370S has persisted at high frequency because it conferred a selective advantage or solely because of multiple, severe population bottlenecks (29–32). There has been debate over the decades about how a disease allele could persist at a relatively high frequency in the Ashkenazi Jewish population when it is obviously detrimental (29, 61). The observation that other genetic lysosomal disorders (e.g., Tay–Sachs and Niemann–Pick) are also found at elevated frequency in this population suggested the possibility of positive selection of these detrimental alleles, possibly from a heterozygote advantage (29, 30, 33, 61, 62).

TB has been cited as the potential selective force amid speculation that densely populated urban living conditions in ghettos might have placed Jews under stronger selection than other Europeans (29, 30, 61–63). TB was a significant killer of young people in Europe through the Middle Ages into the 19th century (64, 65). Indeed, an estimate of mortality based on historical records from 1891 to 1900 attributed half of all deaths in the reproductive years (ages 25 to 40 y) to TB, calculated to lead to a loss of 7 to 15% of reproductive fitness per generation compared to a hypothetical cohort of completely TB-resistant individuals (64). In contrast, even untreated Gaucher disease caused by N370S homozygosity likely has a far lower reproductive fitness cost for the following reasons: 1) half to two-thirds of individuals are asymptomatic (29, 51), and 2) even in those individuals who are symptomatic of Gaucher disease–associated pathologies, fertility is normal (16, 25, 52, 66).

It is difficult to determine the exact age of the N370S allele owing to confounding variables such as admixture (67, 68), uncertain mutation and recombination rates, and conflicting data on historical population sizes (32). Nevertheless, all estimates based on haplotype analyses agree that it is at least 800 y old, with some finding it to be as much as 1,400 y old (32, 69, 70). Therefore, N370S would have been present in the Ashkenazim through the centuries when their exposure to TB would have been high. If individuals homozygous for GBA N370S were more resistant, they would have had a substantial survival and reproductive advantage during periods of high TB transmission. We argue therefore for a model of homozygote protection against TB as a selective force rather than or in addition to the generally accepted “balanced polymorphism” concept of heterozygote advantage offsetting the deleterious effects of homozygosity (29–32, 71). Given the broad antibacterial activity of glucosylsphingosine, it is intriguing to speculate that GBA N370S homozygotes might have also been protected against common but consequential bacterial infections other than TB, contributing additionally to the selective advantage of this allele.

## Materials and Methods

Materials and methods are fully described in *SI Appendix*, *Materials and Methods*. Detailed therein are zebrafish husbandry procedures and methods pertaining to manipulation and assessment of 1) zebrafish larvae: husbandry, drug administration and infection, zebrafish and human mRNA expression and construction of transgenic lines, microscopical assessment of infection and macrophage morphology, and (glyco)sphingolipid and enzyme activity analysis, and 2) bacteria: assessment of drug MICs and minimum bactericidal concentrations (MBCs) and negative stain TEM. Statistical analyses were performed using Prism 9 (GraphPad). Zebrafish lines, plasmids and oligonucleotides used to generate and identify zebrafish lines, and bacterial strains used are listed, respectively, in *SI Appendix,* Tables S1 to S3. Zebrafish husbandry and experimental procedures were conducted in compliance with guidelines from the UK Home Office.

## Supplementary Material

Appendix 01 (PDF)

Movie S1. Swimming abnormality (spinning) observed in 2.5 month-old *gba1^sa1621/sa1621^* mutant animals compared to normal swimming of their wild-type siblings. Of the four fish in the tank, the larger two are wild-type and the smaller two are *gba1^sa1621/sa1621^* mutants.

## Data Availability

All study data are included in the article and/or *SI Appendix*.
