# Supplementary material for: Gaucher Disease Protects Against Tuberculosis
Source: Proc Natl Acad Sci U S A. Author manuscript; Available in PMC 2023 Feb 24. (PMC7614233; doi:10.1073/pnas.2217673120)
Supplement: Supplementary file 1 [file EMS170516-supplement-Supplementary_Material.pdf]

Supporting Information for
Gaucher Disease Protects Against Tuberculosis

Jingwen Fan[1,2], Victoria L. Hale[2#], Lindsey T Lelieveld[3#], Laura J. Whitworth[1,2], Elisabeth M. Busch-Nentwich[1,4], Mark Troll[1,2], Paul H. Edelstein[1, 5], Timothy M. Cox[6], Francisco J. Roca[1,7], Johannes M.F.G. Aerts[3] and Lalita Ramakrishnan[1,2*]

[#]Equal contributors

Corresponding author: Lalita Ramakrishnan
Email: lalitar@mrc-lmb.cam.ac.uk

This PDF file includes:

Supporting text
Tables S1 to S3
Box S1
Figures S1 to S8
Legends for Movies S1
SI References

Supporting text

Methods

Zebrafish husbandry

Zebrafish husbandry and experimental procedures were conducted in compliance with guidelines from the UK Home Office. All zebrafish were maintained in buffered reverse osmotic water systems and were exposed to a 14-hour light -10-hour dark cycle to maintain proper circadian conditions. They were fed twice daily a combination of dry food and brine shrimp. The *gba1* mutant fish were culled at 2.5 months old as soon as clinical signs of disease were observed.

Fish Lines

The mutant lines *gba1*^{sa1621} and *asah1b*^{sa19461} were created by ENU (N-ethyl-N-nitrosourea) mutagenesis as part of The Zebrafish Mutation Project (ZMP) (1) and provided by the Wellcome Sanger Institute. The allele *gba1*^{sa1621} has been previously used to study larval bone ossification (2). The *gba1*^{sa1621}and *asah1b*^{sa19461} lines were generated in the Tupfel long-fin (TL) strain and outcrossed to the AB wild-type strain (Zebrafish International Resource Center) to be maintained in a mixed AB/TL background. The *gba1*^{cu41} and *gba1*^{cu42} mutant lines and the transgenic lines *Tg(mpeg1:YFP)*^{w200} (with YFP-expressing macrophages) (3) and *Tg(mpeg1:Brainbow)*^{w201} (with tdTomato-expressing macrophages) (4) were created and maintained in the AB background. The transgenic zebrafish lines *Tg(BH:eGFP-mfap4:hWT)*^{cu57}, *Tg(BH:eGFP-mfap4:N370S)*^{cu58}, and *Tg(BH:eGFP-mfap4:L444P)*^{cu59}, were generated and maintained as described before (5); briefly, the human wild-type *GBA1* cDNA (hWT) (Sino Biological, NM_000157) was amplified by Phusion High-Fidelity (NEB) PCR. The 3´ adenine (A) overhangs were then added by Taq (NEB) PCR and cloned into the TOPO-TA plasmid (Thermo Fisher Scientific). The human wild-type *GBA1*-containing TOPO-TA plasmid was then used as a template to generate the N370S and L444P *GBA1* variants, using the Q5® Site-Directed Mutagenesis Kit (NEB). Then, each version was inserted after the macrophage-specific mfap4 promoter into the plasmid pTol2-PhiC31LS-BH:GFP-mfap4:New MCS and these plasmids were used to generate the transgenic lines (5). G0 *gba1*-deficient zebrafish embryos (crispants) were generated using CRISPR-Cas9 technology by simultaneously targeting different sites of the *gba1* gene (6). Guide RNAs were prepared following the manufacturer specifications, hybridizing the common RNA component (Alt-R tracrRNA) with each of the specific Alt-R crRNA. 3-5 nl of a solution containing Alt-R crRNA and Alt-R tracrRNA (30 µM each) complexed with Cas9 protein (0.25 µg/µl) (Integrated DNA Technologies), and 2% phenol red sodium salt (Sigma) was injected into 1-2 cell stage embryos (6). Similar volumes of a solution containing Cas9 protein and phenol red was used to generate the control animals. The genotype of individual larvae and mutagenesis efficacy were assessed by high-resolution melt (HRM) analysis (7). The mutant lines *gba1*^{cu42} and *gba1*^{cu41} were generated by targeting the *gba1* gene with Alt-R crRNA3 (the same crRNA3 as used for the G0 crispants) and Alt-R crRNA5. Genotypes were determined by high-resolution melt analysis (HRM) (7). Founders were identified in the F1 generation and the lines were outcrossed to the AB wild-type strain.

Bacterial strains

Mycobacterium marinum (Mm) M strain (ATCC #BAA-535) expressing tdTomato (red fluorescence), mWasabi (green fluorescence) or EBFP2 (blue fluorescence) under the *msp12* promoter (8) were grown under hygromycin (Mediatech) selection in Middlebrook 7H9 medium (Difco), supplemented with oleic acid-albumin-dextrose-saline (OADS), and Tween-80 (Sigma) (8) at 33ºC. *Mycobacterium smegmatis* (Msm)(mc^2 155) was grown in Middlebrook 7H9 medium supplemented with albumin-dextrose-catalase (ADC) at 37ºC (9). *M. tuberculosis* (Mtb) H37Rv *ΔleuD ΔpanCD* double auxotroph (mc^2 6206) expressing tdTomato under the *msp12* promoter was grown under hygromycin B selection in Middlebrook 7H9 broth supplemented with oleic acid-albumin-dextrose-catalase (OADC), Tween-80 (Sigma), 0.05 mg/ml L-leucine and 0.024 mg/ml calcium pantothenate (Sigma) at 37ºC (5, 10). Single cell suspensions were prepared of all mycobacterial strains to infect zebrafish and for MIC/MBC tests.(8).

Staphylococcus aureus (ATCC 29213) and *Escherichia coli* (ATCC 25922) were passaged twice on tryptic soy agar with 5% sheep's blood (Thermo Scientific) at 37ºC in 5% CO_2 for 18-24 hours.

3 to 5 isolated morphologically normal colonies selected from the agar plates were suspended in cation adjusted Mueller Hinton broth (CAMHB, Sigma-Aldrich). The bacterial suspensions were diluted with normal saline to achieve a turbidity equivalent to a 0.5 McFarland standard (latex, Thermo Scientific) using either a Wickerham card (Thermo Scientific) or by nephelometry (DensiCHEK Plus, bioMérieux) before drug exposure.

Assessment of clinical signs of disease in adult *gba1* deficient zebrafish

As soon as clinical signs of disease were observed, photographs and videos of adult *gba1* zebrafish were taken using the camera incorporated within a cell phone (Huawei P10). Immediately thereafter, the animals were euthanized by tricaine overdose followed by brain destruction according to Schedule 1 of the Animals (Scientific Procedures) Act 1986. Genotypes were confirmed post-mortem.

Assessment of drug minimum inhibitory concentrations (MIC) and minimum bactericidal concentrations (MBC)

Glucosylsphingosine (Sigma-Aldrich) and D-erythro-Sphingosine (C18) (Santa Cruz Biotechnology) were dissolved in absolute ethanol (Honeywell) at 6 mM. Glucosylceramide (GlcCer, Sigma-Aldrich) was dissolved in DMSO-ethanol (1:1, v/v) at 20 mM. Rifampicin (Sigma) was dissolved in methanol (Honeywell) at 25 mg/ml. Aliquoted drugs were stored at −20ºC and used within a month.

All MIC/MBC tests were performed according to CLSI instructions (M07-11th edition for broth MICs and M26A-1999 for bactericidal determination) (11, 12) with modifications as specified below for mycobacterial testing. On the day of the MIC/MBC assessment, all drugs were diluted in broth media twofold to achieve the desired test concentrations. Liquid broth containing 4% absolute ethanol or 4% DMSO-ethanol (1:1, v/v) showed no inhibition of bacterial growth. 100 µl of liquid broth containing varying concentrations of each drug were added in duplicate into sterile round bottom polystyrene 96-well plates (Corning). The inocula for *S. aureus* (ATCC 29213) and *E. coli* (ATCC 25922) were prepared by making a 1:100 dilution in CAMHB of the 0.5 McFarland standard equivalent turbidity of the bacterial culture. Mycobacterial inocula were prepared by diluting single cell suspensions to OD_{600} 0.03. 100 µl of bacterial culture (without Tween-80) was added to the previously prepared plate containing 2X final drug concentrations. The final bacterial inoculum of approximately 5 x10^5 cfu/ml in the MIC/MBC test was confirmed by counting CFU on agar plates.

Drug-treated *S. aureus* and *E. coli* microbroth plates were incubated at 37ºC (5% CO_2) for 16-20 hours without shaking. Drug-treated Mm microbroth plates were incubated at 33ºC for 5-7 days without shaking. Drug-treated Msm was incubated at 37ºC for 2-3 days without shaking. Drug-treated Mtb was incubated at 37ºC for 14-21 days in 5% CO_2 without shaking. The MIC was defined as the lowest concentration of drug which completely inhibited bacterial growth as detected by eye. According to CLSI instructions, the MIC of the quality control drug rifampicin should be: 0.004 to 0.016 mg/L for *S. aureus*, and 4 to 16 mg/L for *E. coli*. The MBC was defined as a $\geq 3\log_{10}$ decrease from the initial concentration by plating drug treated bacterial culture with ten-fold serial dilutions after the MIC was determined. *S. aureus* and *E. coli* were plated on CAMHB media at 37ºC overnight. Mm was plated on 7H10+OADS media at 33ºC for 7-9 days. Msm was plated on 7H10+ADC media at 37ºC for 2-3 days. Mtb was plated on 7H10+OADC+leu+pan media at 37ºC and grown in a humidified incubator with 5% CO_2 for a month. Drug carryover inhibiting bacterial growth on the culture plates was excluded because the ten-fold dilutions resulted in drug concentrations below their respective MICs for the test organisms.

Zebrafish infections

Zebrafish embryos were housed in fish water (reverse osmosis water containing 0.18 g/l Instant Ocean supplemented with 0.25 mg/ml methylene blue) up to 1 day post-fertilization (dpf) at 28.5°C. From 1 dpf, embryos were maintained in 0.5X E2 medium (13) with 0.003% PTU (1-phenyl-2-thiourea, Sigma).

To assess bacterial burdens, zebrafish were infected with Mm at 2 dpf in the hindbrain ventricle (HBV) or the caudal vein (CV) (8). Inocula of 200–300 and 100–150 Mm were used respectively, for CV and HBV. Mtb bacterial burdens were assessed after CV infection using inocula of 400–800 bacteria. For infections using a heterozygotes incross to generate all genotypes, larvae were genotyped using KASP ($gba1^{sa1621}$ and $asah1b^{sa19461}$) or HRM ($gba1^{cu41}$ and $gba1^{cu42}$) assays at the end of experiment.

For infectivity assays, highly diluted mWasabi-expressing Mm (0-3 cfu/nl) or tdTomato-expressing Mtb (0-3 cfu/nl) were injected into the HBV at 2 dpf. Staining with pHrodo Red to assess phagosomal versus phagolysosomal localization of the bacteria was performed before infection into the HBV as described (14). The phagosome- and phagolysosome-located Mm were sorted 12-24 hours post infection by confocal microscopy.

Microscopy

Assessment of macrophage morphology and speed

To assess macrophage vacuolation, 1 mM LysoTracker Red DND-99 (Life technologies) in DMSO solution was diluted 1:25 in PBS and injected into the CV of 3 dpf zebrafish larvae. Larvae were then incubated at 28.5°C in the dark for 1 hour before confocal imaging. To assess engulfment accumulation of cell debris in macrophages, acridine orange (ImmunoChemistry, Bloomington, MN) was dissolved in 0.5X E2 fish water at 2 µg/mL and larvae were soaked in this solution at 28.5°C in the dark for 30 minutes before imaging. Macrophage morphology was assessed using a Nikon A1R confocal microscope with a 20X Plan Apo 0.75 NA objective with a galvano scanner used to generate 40 µm *z*-stacks consisting of 1.3–2 µm optical sections. Images were acquired and processed with NIS Elements (Nikon) using the Denoise.ai function and maximum intensity projection. Macrophage, LysoTracker and acridine orange volumes were measured using the surface rendering feature of Imaris 9.1 (Bitplane Scientific Software).

For macrophage speeds, using the same settings as above, timelapse images were taken using a resonant scanner at 3-minute intervals for 2 hours maintaining the larvae at 28.5°C using a heating chamber (Okolab). Macrophage tracks were generated by surface rendering and object classification features of Imaris 9.7 (Bitplane Scientific Software), and the mean speed of each track calculated. When the fish used in experiments were generated from *gba1* heterozygote incrosses, their genotype was determined by KASP or HRM assay at the end of experiment, ensuring blinded quantification and analysis.

Assessment of infection

Bacterial burdens were assessed 3 dpi for HBV infections and 5 dpi for CV infections. To assess bacterial burdens in HBV infections, zebrafish larvae were anesthetized in 0.5X E2 media containing 0.025% Tricaine and embedded in 2.0% low melting point agarose on 6-well optical bottom plates (MatTek Corporation) before imaging. For CV infections, animals were anesthetized in 0.025% tricaine (Sigma-Aldrich) before imaging. Bacterial burdens were assessed by fluorescence pixel counts (FPC) using wide-field microscopy was performed using a Nikon Eclipse Ti-E equipped with a C-HGFIE 130W LED light source and 4X objective. Fluorescence images were captured with a CoolSNAP HQ2 Monochrome camera (Photometrics) using NIS-Elements (version 3.22). Fluorescence filter cubes sets included Chroma FITC (41002), Cy3/TRITC (41004), for detection of green and red fluorescence, respectively.

For HBV infection, Mm volume was assessed using a Nikon A1R confocal microscope with a 20X Plan Apo 0.75 NA objective with a galvano scanner used to generate 40 µm *z*-stacks consisting of 1.3–2 µm optical sections. Images were acquired and processed with NIS Elements (Nikon) using the Denoise.ai function and maximum intensity projection. After HBV infection, bacterial volumes were measured using the surface rendering feature of Imaris 9.1 (Bitplane Scientific Software).

For the infectivity assays, as soon as all the animals had been infected, larvae that had been infected with a single bacterium were identified as follows: the larvae were gently embedded in 3% methylcellulose in 6-well optical bottom plates (MatTek Corporation) in a supine position and the number of infecting bacteria counted using a Nikon Eclipse Ti-E inverted microscope with a 10 or 20X objective and 10X ocular. At 5dpi, the larvae were assessed for the presence or absence of bacteria using a Nikon Eclipse Ti-E inverted microscope with a 10X or 20X objective and 10X ocular.

Drug administration to zebrafish larvae

Carmofur (Abcam) was dissolved in DMSO (Sigma) and stored in small aliquots at 50 mM at −20°C. 1 dpf embryos were treated fish water with 0.5 μM carmofur (Abcam) with the corresponding volume of 1% DMSO used for the control group. The fish water was renewed daily with freshly prepared drug until the end of the experiment.

To administer VPRIV® (velaglucerase alfa), 2 dpf larvae infected in the HBV were randomly distributed into control and velaglucerase-treated groups at four hours post-infection and 1-1.5 nl PBS containing velaglucerase alfa (100 units/ml) or PBS alone injected into the HBV.

RNA and morpholino injections

Total RNA was extracted from 14 dpf wild-type zebrafish and reverse transcribed into cDNA with PrimeScript™ 1st strand cDNA Synthesis Kit (Takarabio). The zebrafish wild-type cDNA and human wild-type *GBA1* (Sino Biological, NM_000157) were amplified by Phusion polymerase (NEB) PCR with a forward primer containing the sequence for the T7 promoter (5'-TAATACGACTCACTATAGG-3') followed by a Kozak sequence (5'-GCCGCCACC-3'). The 3´ adenine (A) overhangs were then added by Taq PCR (NEB) and cloned into the TOPO-TA plasmid (Thermo Fisher Scientific) and cloned into the TOPO-TA plasmid (Thermo Fisher Scientific).

The human wild-type *GBA1* TOPO-TA plasmid was used as a template to generate the N370S and L444P *GBA1* variants, using the Q5® Site-Directed Mutagenesis Kit (NEB). Primers designed with 5' ends annealing back-to-back (NEB online design software NEBaseChanger™) were used to produce all *GBA1* variants.

The mMESSAGE mMACHINE™ T7 Transcription Kit (Thermo Fisher Scientific) followed by Poly(A) Tailing Kit (Thermo Fisher Scientific) was used for *in vitro* mRNA production. 3-5 nL of a solution containing each RNA (200 ng/ul and 300 ng/ul for zebrafish and human, respectively), or 0.5 mM *gba1* morpholino (Gene Tools), 0.5X Tango Buffer (Thermo Scientific), and 2% phenol red sodium salt (Sigma) was injected into the yolk of 1-2 cell-stage embryos.

Negative stain-transmission electron microscopy (TEM)

For TEM experiments, bacteria were treated with drugs as for the MIC/MBC experiments. CFU counts were determined by plating before and after drug treatments. Bacterial cultures were spun down and gently washed twice with a 10 mM Tris–HCl solution containing 5 mM glucose (MRC LMB Media and Glass Wash) and a final concentration of 2 x 10^7-2 x10^8 cfu/ml bacteria (OD_{600}=0.1-1) was used to prepare the electron microscopy grids.

For negative staining, carbon-coated 300-mesh electron microscopy grids (Agar Scientific) were glow discharged just prior to use (PELCO easiGlow; 45 seconds at 15-25 mA). Grids were incubated for 1 minute with 3 μL bacterial culture, prepared as above, then side-blotted to remove excess. A 3 μL drop of 0.75% uranyl formate was applied to the grid and immediately side-blotted, before the addition of a second 3 μL drop of 0.75% uranyl formate. This was incubated for 30 seconds before side blotting as before. Grids were allowed to dry completely before TEM imaging. TEM imaging was carried out at 120 keV on a ThermoFisher (Formerly FEI) Tecnai G2 Spirit equipped with Gatan Orius SC200W CCD camera. For quantification, grid squares with good staining were randomly selected and every cell in those squares was imaged.

(Glyco)sphingolipid analysis

Lipids were extracted and analyzed from 5 dpf individual zebrafish larvae as described previously (15, 16).

Assessment of Gba1 activity

β-glucocerebrosidase (GCase) activity was measured as described earlier (17), using homogenates prepared from pools of 5 dpf zebrafish (10 zebrafish larvae per sample) in potassium phosphate lysis buffer containing: 25 mM K_2HPO_4-KH_2PO_4 pH 6.5, 0.1% (v/v) Triton-X100 and EDTA-free protease inhibitor (cOmplete™, EDTA-free Protease inhibitor Cocktail, Roche, Sigma-Aldrich). All activities were measured using three independent homogenates in technical duplicate.

Statistical Analysis
The following statistical analyses were performed using Prism 9 (GraphPad): Student's unpaired t-test, one-way ANOVA with Tukey's post-tests and Fisher's exact test. *$P < 0.05$; **$P < 0.01$; ***$P < 0.001$; $P < 0.0001$; not significant, $P \geq 0.05$.

Table S1. Zebrafish lines used in this study.

Zebrafish lines	Source	Identifier
Zebrafish (*Danio rerio*): wild type AB strain	Zebrafish International Resource Center	ZFIN ID: ZDB-GENO-960809-7
Zebrafish: *Tg(mpeg1:YFP)*^{w200}	(3)	ZFIN ID: ZDB-FISH-150901-6828
Zebrafish: *Tg(mpeg1:Brainbow)*^{w201}	(4)	ZFIN ID: ZDB-FISH-151204-7
Zebrafish: *gba1*^{sa1621}	The Zebrafish Mutation Project (ZMP) at the Wellcome Sanger Institute	ZFIN ID: ZDB-GENE-100922-270
Zebrafish: *asah1b*^{sa19461}	ZMP at the Wellcome Sanger Institute	ZFIN ID: ZDB-GENE-040426-1512
Zebrafish: *gba1*^{cu41}	This paper	University of Cambridge Zebrafish line ID: cu41
Zebrafish: *gba1*^{cu42}	This paper	University of Cambridge Zebrafish line ID: cu42
Zebrafish: *Tg(BH:eGFP-mfap4:hWT)*^{cu57}	This paper	University of Cambridge Zebrafish line ID: cu57
Zebrafish: *Tg(BH:eGFP-mfap4:N370S)*^{cu58}	This paper	University of Cambridge Zebrafish line ID: cu58
Zebrafish: *Tg(BH:eGFP-mfap4:L444P)* ^{cu59}	This paper	University of Cambridge Zebrafish line ID: cu59

Table S2. Plasmids and oligonucleotides used in this study.

Plasmids or oligonucleotides	Source	Identifier
TOPO-TA plasmid	Thermo Fisher Scientific	CAT# K4575J10
pCMV3-untagged-human *GBA1* cDNA ORF plasmid	Sino Biological	CAT# HG12038-UT
pTol2-PhiC31LS-BH:GFP-mfap4:New MCS	(5)	N/A
pTol2-PhiC31LS-BH:GFP-mfap4:hWT	This paper	N/A
pTol2-PhiC31LS-BH:GFP-mfap4:N370S	This paper	N/A
pTol2-PhiC31LS-BH:GFP-mfap4:L444P	This paper	N/A
Guide RNA 1 sequence for G0 CRISPR: 5'-CATACTGCGACTCTCTCGGC-3'	This paper	crRNA1
Guide RNA 3 sequence for G0 CRISPR: 5'-GAGTGCTGGACCTAGATCCA-3'	This paper	crRNA3
Guide RNA 4 sequence for G0 CRISPR: 5'-GGGTGACGTCAGGCGGCACA-3'	This paper	crRNA4
Guide RNA 5 sequence for *gba1^{cu41}* creation: 5'-GGATCGTGGTGTGCGTCT-3'	This paper	crRNA5
Forward primer sequence for genotyping by HRM of G0 crispant created by guide RNA 1: 5'-GGTCATGGCTCGGTTGTGT-3'	This paper	*gba1* G0 Forward-1
Reverse primer sequence for genotyping by HRM of G0 crispant created by guide RNA 1: 5'-CTCCATCAGTCTGCTGCCAG-3'	This paper	*gba1* G0 Reverse-1
Forward primer sequence for genotyping by HRM of G0 crispant created by guide RNA 3: 5'-AGGCTTTGGGTTTTACTCCG-3'	This paper	*gba1* G0 Forward-3
Reverse primer sequence for genotyping by HRM of G0 crispant created by guide RNA 3: 5'-TCATTAGTTGGGTCTTGGAAAAG-3'	This paper	*gba1* G0 Reverse-3
Forward primer sequence for genotyping by HRM of G0 crispant created by guide RNA 4: 5'-CACGATATGTCCACGGCATT-3'	This paper	*gba1* G0 Forward-4
Reverse primer sequence for genotyping by HRM of G0 crispant created by guide RNA 4: 5'-GGAAGTAATCAGGGTACAGATGGT-3'	This paper	*gba1* G0 Reverse-4
Forward primer sequence for genotyping by HRM of *gba1^{cu41}* created by guide RNA 5: 5'- CCATCTGTACCCTGATTACTTCC-3'	This paper	*gba1^{cu41}* Forward-5
Reverse primer sequence for genotyping by HRM of *gba1^{cu41}* created by guide RNA 5: 5'-TTAAGACCATTATCCAGTACCTGG-3'	This paper	*gba1^{cu41}* Reverse-5
Forward primer sequence to amplify human *GBA1* cDNA: 5'-ATGGAGTTTTCAAGTCCTTCCAGAG-3'	This paper	hGBA1-F
Reverse primer sequence to amplify human *GBA1* cDNA: 5'-TCACTGGCGACGCCACAG-3'	This paper	hGBA1-R
Forward primer sequence to produce human *GBA1-N370S* DNA by Q5® Site-Directed Mutagenesis Kit: 5'-ATCATCACGAgCCTCCTGTAC-3'	This paper	N370S-Q5-F
Reverse primer sequence to produce human *GBA1-N370S* DNA by Q5® Site-Directed Mutagenesis Kit: 5'-GCTGTGGCTGTACTGCAT-3'	This paper	N370S-Q5-R

Forward primer sequence to produce human *GBA1-L444P* DNA by Q5® Site-Directed Mutagenesis Kit: 5'-AAGAACGACCcGGACGCAGTG-3'	This paper	L444P-Q5-F
Reverse primer sequence to produce human *GBA1-L444P* DNA by Q5® Site-Directed Mutagenesis Kit: 5'-CTGACTGGCAACCAGCCC-3'	This paper	L444P-Q5-R
gba1 Morpholino: 5'-CGTCTGATTATTGTTACACACCTCT-3'	(18)	*gba1* MO

Table S3. Bacterial strains used in this study.

Bacterial strains	Source	Identifier
Mycobacterium marinum M strain transformed with pMSP12:tdTomato	(8)	derivatives of ATCC #BAA-535
M. marinum M strain transformed with pMSP12:wasabi	(8)	derivatives of ATCC #BAA-535
M. marinum M strain transformed with pMSP12:EBFP2	(8)	derivatives of ATCC #BAA-535
M. tuberculosis ΔleuD ΔpanCD double auxotroph strain transformed with pMSP12: tdTomato	Gift from Bill Jacobs' Laboratory	derivatives of mc^2 6206
M. smegmatis	American Type Culture Collection (ATCC)	mc^2 155
Staphylococcus aureus	ATCC	ATCC 29213
Escherichia coli	ATCC	ATCC 25922

Supplementary Box S1

Box 1. Calculation of Glucosylsphingosine Concentrations in Gaucher Disease

MACROPHAGES

Human macrophage glucosylsphingosine (GS) concentrations were calculated using published data, which found ~ 0.6 nanomole per mg protein (19, 20). To convert this into a concentration, we used the number of moles per gram of cells, and separately, the volume of water per gram of cells. Dividing the first quantity by the second gives the number of moles of GS per volume of water in the cells, or the cellular concentration in moles/liter.

$$\frac{mole\_GS}{g\_cells} \Big/ \frac{Vol\_Water\_in\_cells}{g\_cells} = concentration[GS]$$

Since about 20% of a cell's mass is protein (21), we have:

$$\frac{mole\_GS}{g\_cells} = \frac{mole\_GS}{g\_protein} \times \frac{g\_protein}{g\_cells} = \frac{mole\_GS}{g\_protein} \times 0.2$$

With a typical cell density (ρ) of 1.1 g/cm^3 (1100 g/Liter) (22), and about 70% of the cell volume being water (23):

$$\frac{Vol\_Water\_in\_cells}{g\_cells} = \frac{Vol\_cells}{g\_cells} \times \frac{Vol\_Water\_in\_cells}{Vol\_cells}$$

$$= \frac{1}{\rho} \times 0.7 = (6.36E - 4) Liters/g\_cells$$

Therefore, 0.6 nanomoles per mg of cells (=0.6 micromoles/g) would correspond to:

$$6E - 7(mole/g) \times (0.2/6.36E - 4) = (1.89E - 4)M = 189\,\mu M$$

SPLEEN

Concentrations in spleen were calculated from published values for four patients with Type 1 Gaucher disease (11.8, 7.8, 15.6 and 17.8 mol/kg), estimating water content to be 70% of total weight (23).

NOTES ON CALCULATION

The amount of "free" water in a cell is much less than the total water determined by the difference between dry and wet weight [this is the typical method of determining water content]. Much of the water (~ 25%) in cells is strongly associated with proteins, salts, small molecules, and the headgroups of membranes. So calculations based on the total amount of glucosylsphingosine and the total amount of water use a simplistic model and can only be interpreted as a plausibility rationale. Since it is certain that the available water is less than the total, the concentration of GS will be higher than calculated. The estimate provided can therefore be interpreted as a lower limit. While the estimates for water content, density, and protein content are rough, they have no effect on "order of magnitude" calculations. Given the uncertainties, it is inappropriate to use more than one or two significant figures in reporting estimated concentrations (e.g., ~200 μM rather than 189 μM).

A

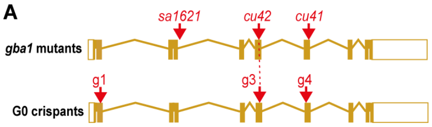

B

Allele	cDNA RT-PCR sequencing result	Translated amino acid sequence	Mutation type
sa1621	WT AAAGAGGATGTCCACATGAAGAT------------------------------------<br>sa1621 AAAGAGGATGTCCACATGAAGATAGCATACACAAACAAAAACACACCTTGCTACAATTAG<br>WT ----------------------------TCCCCTTCTGCAGCGGGCACAGGCTCTCTCTG<br>sa1621 CGGTCTGCAGTTGTACTCGCTTAAGGATTCCCCTTCTGCAGCGGGCACAGGCTCTCTCTG	I E Y R F V R V P V A S C D F S T R L Y T Y A D T<br>P E D Y D L Q N F T L A K E D V H Met K I A Y P<br>C Y N **Stop** R S A V V L A **Stop** G F P F C S G H<br>R L S L L S L S I C S P V P G A P P P G **Stop** R P T<br>A H **Stop Stop** G R A P S K A N Q E A K N T R P<br>G L S T I S	Frame-shift
cu41	WT GCGCTGGGTGGAGTCCAGTGGATCGTGGTGTGCGTCTGGGCAGCTGGGACAGGGCAGAGG<br>cu41 GCGCTGGGTGGAGTCCAGTGGATCGTGG--------TGGGCAGCTGGGACAGGGCAGAGG	**A** L H S S S F S K T Q L **Met** I L D D N R L **Met** L<br>P H W A K V V L S D I K A A R Y V H G I G V H<br>W Y F D R L A P P D V T L T S T H H L Y P D Y F<br>L F A T E A C A G W S P V D R G G Q L G Q G R<br>G L R P **Stop** **H** H P G P E **Stop** **L** C D R L D R L E<br>F G P **Stop** **S** R W R T Q L G Q E L	Frame-shift
cu42	WT GACTGGATTGCCCTGG-----------------ATCTAGGTCCAGCACTCCATTCCTCAT<br>cu42 GACTGGATTGCCCTGGATAATAATATACGTTGAATCTAGGTCCAGCACTCCATTCCTCAT	K I P L L Q R A Q A X S A Q P L Y X F A S A W S A<br>P A W L K T N G A L I G K G S L K G K P G G K E H<br>K T W A Q Y Y I R F L E E Y R K Y N L S F W G L T<br>S G N E P T A G E Met T N Y S F Q A L G F T P E T<br>Q R D W I A L D N N I R Stop I Stop V Q H S I P H<br>P F P R P N Stop Stop F L	Frame-shift

Fig. S1. *gba1* mutations characterization. *(A)* Diagram of the zebrafish *gba1* gene showing the locations of the three zebrafish mutations used in this study. *(B)* cDNA sequences of the three mutations and predicted amino acid sequence.

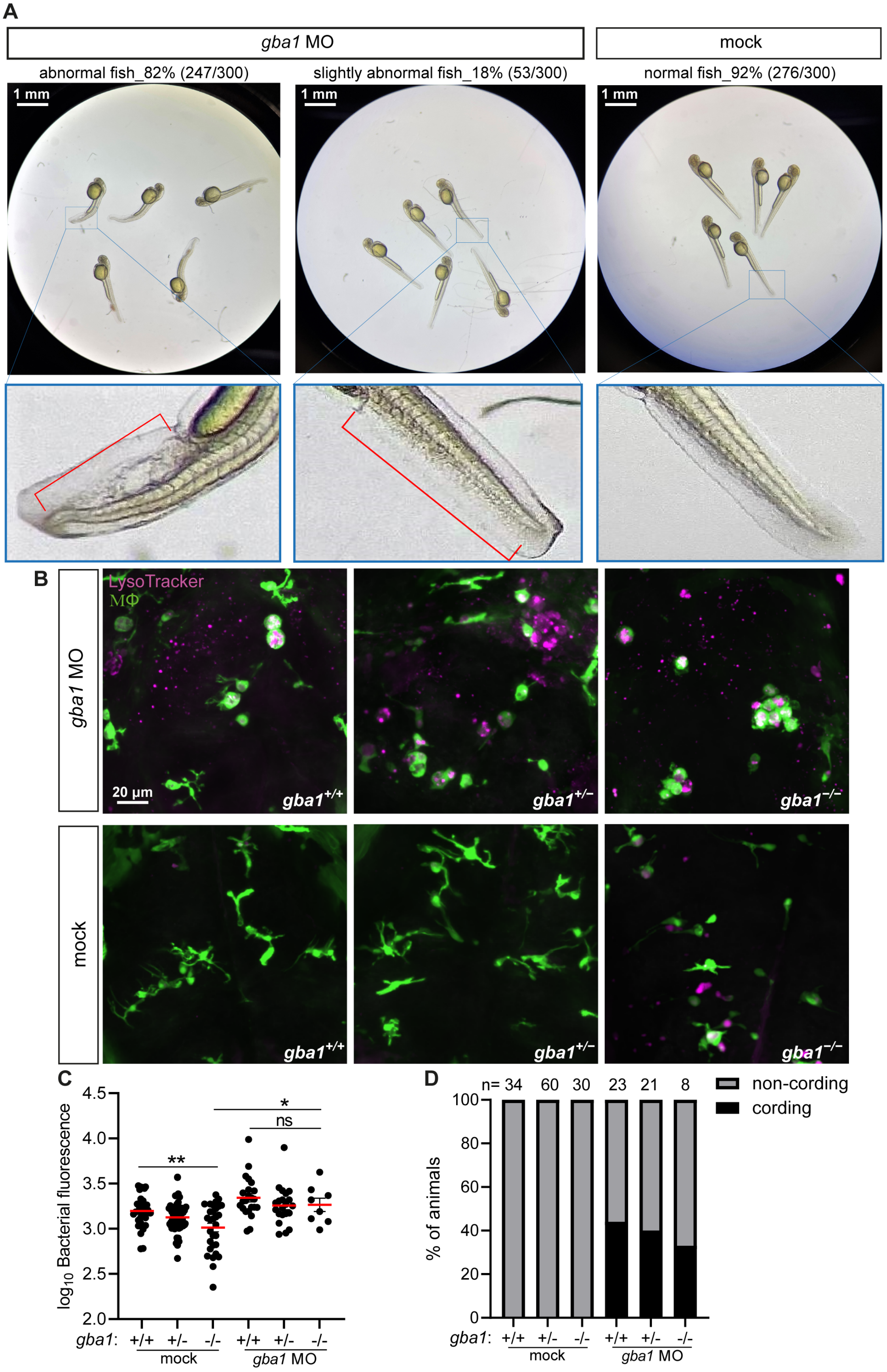
A
gba1 MO
mock
abnormal fish_82% (247/300)
slightly abnormal fish_18% (53/300)
normal fish_92% (276/300)
1 mm
B
LysoTracker
MΦ
gba1 MO
mock
20 µm
gba1+/+
gba1+/–
gba1–/–
C
log10 Bacterial fluorescence
4.5
4.0
3.5
3.0
2.5
2.0
**
*
ns
gba1: +/+ +/- -/- +/+ +/- -/-
mock
gba1 MO
D
n= 34 60 30 23 21 8
non-cording
cording
% of animals
100
80
60
40
20
0
gba1: +/+ +/- -/- +/+ +/- -/-
mock
gba1 MO

Fig. S2. *gba1* Morpholino (MO) induces toxicity. *(A)* Representative images of 2 dpf *gba1* MO-or mock (PBS)-injected fish. Red outline, abnormal jagged blood vessel area. Only slightly abnormal fish from *gba1* MO group were used to assess macrophage morphology and for infection experiments. *(B)* Maximum intensity projection of pseudo-colored representative confocal images of YFP-expressing macrophages stained with LysoTracker Red in 3 dpf zebrafish brains. *(C)* Bacterial burdens in zebrafish larvae of 5 dpi infection into the CV with 200-300 Mm. Horizontal red bars, means; ns, not significant; $^{*}P < 0.05$; $^{**}P < 0.01$ (one-way ANOVA with Tukey's post-test). *(D)* Percentage of zebrafish larvae from *(C)* with extracellular Mm growth (cording). n, number of larvae.

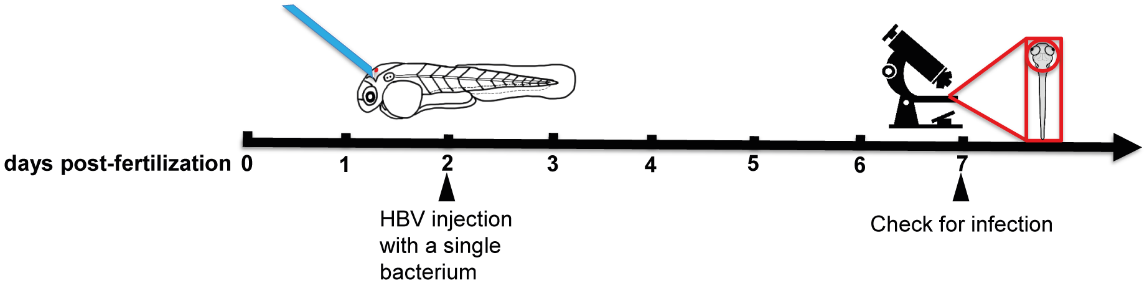

Fig. S3. Schematic diagram of the infectivity experiment.

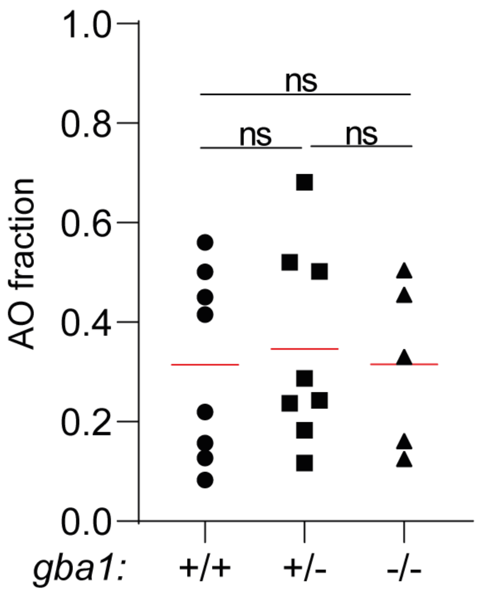

Fig. S4. Quantification of AO volume per macrophage in the caudal hematopoietic tissue (CHT) of 5 dpf zebrafish. Each point represents the average AO volume fraction per macrophage in each fish. Horizontal red bars, means. ns, not significant (one-way ANOVA with Tukey's post-test). Representative of 2 independent experiments.

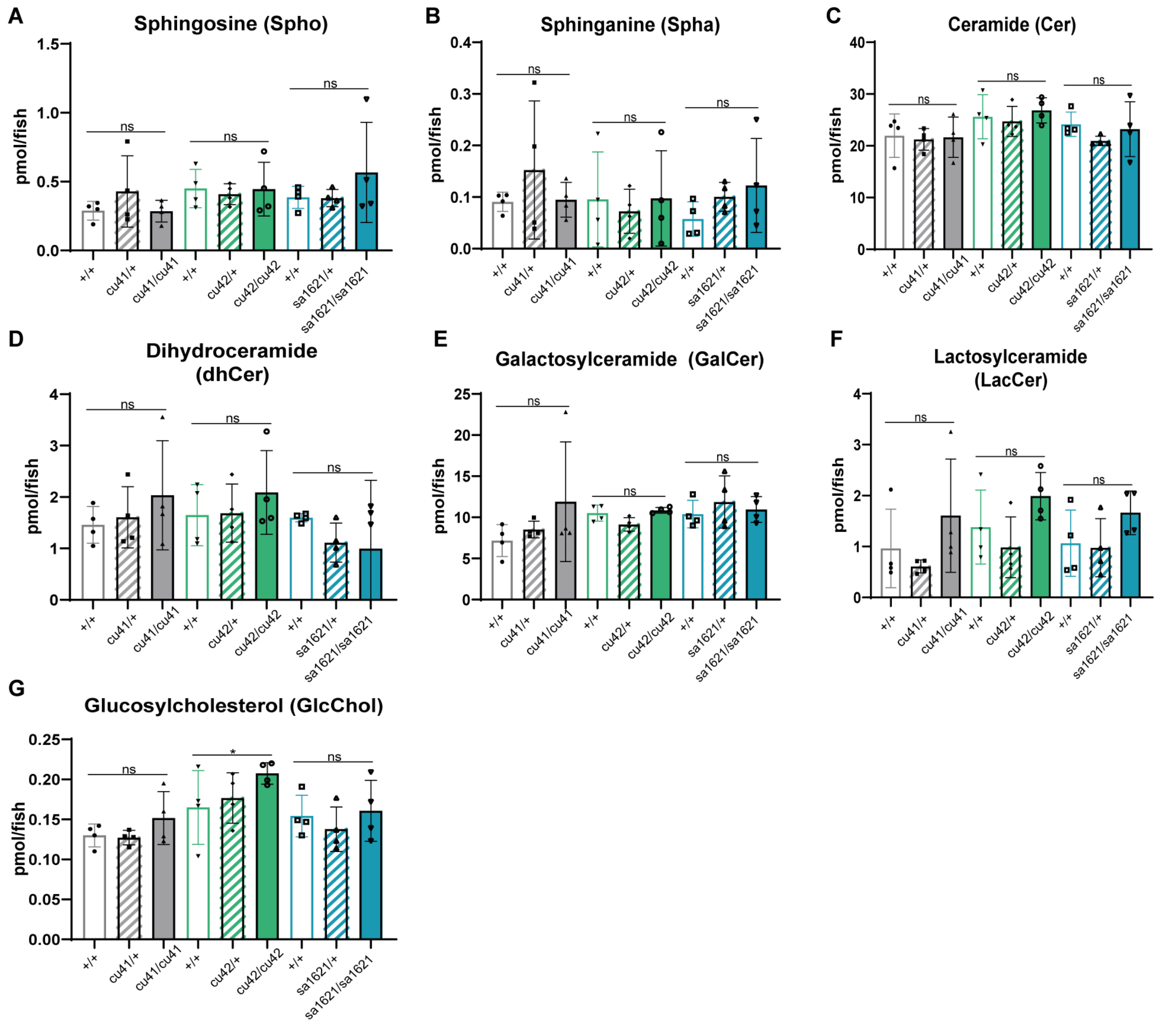

Fig. S5. Quantification of lipids in 5dpf *gba1* mutants and their siblings.

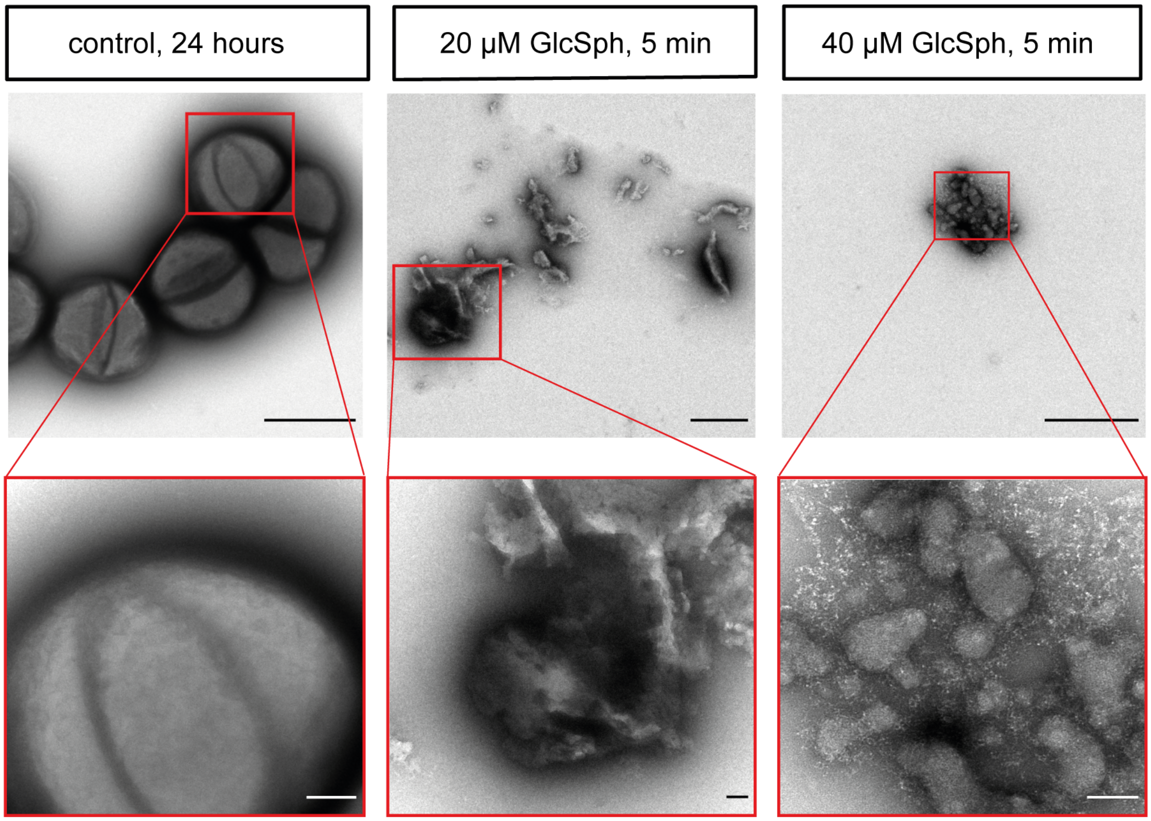

Fig. S6. GlcSph-induced damage to *S. aureus* membranes. Representative negative stain-TEM images of *S. aureus* treated with GlcSph. Scale bar , top panel, 1 µm, bottom panel, 100 nm.

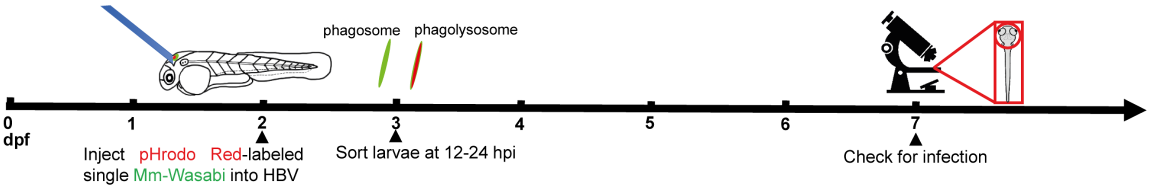

Fig. S7. Schematic diagram of the infectivity experiment in the context of examining the effect of mycobacterial phagosomal versus phagolysosomal localization.

A

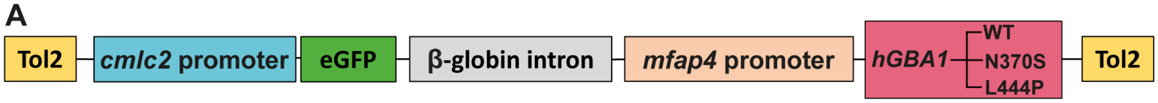

B

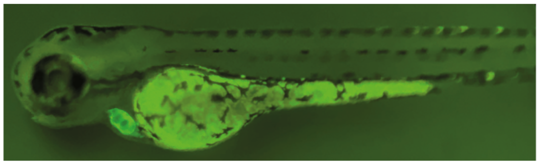

Fig. S8. Transgenic zebrafish expressing human *GBA1* mutations in macrophages. *(A)* Schematic diagram showing the structure of the plasmid to generate the transgenic zebrafish line expressing macrophage-specific human *GBA1* mutations. *(B)* Representative image of the eGFP bleeding heart positive fish.

Movie S1 (separate file). Swimming abnormality (spinning) observed in 2.5 month-old *gba1*$^{sa1621/sa1621}$ mutant animals compared to normal swimming of their wild-type siblings. Of the four fish in the tank, the larger two are wild-type and the smaller two are *gba1*$^{sa1621/sa1621}$ mutants.

SI References

1. R. N. Kettleborough *et al.*, A systematic genome-wide analysis of zebrafish protein-coding gene function. *Nature* **496**, 494 (2013).
2. I. Zancan *et al.*, Glucocerebrosidase deficiency in zebrafish affects primary bone ossification through increased oxidative stress and reduced Wnt/β-catenin signaling. *Human Molecular Genetics* **24**, 1280-1294 (2014).
3. F. J. Roca, L. Ramakrishnan, TNF dually mediates resistance and susceptibility to mycobacteria via mitochondrial reactive oxygen species. *Cell* **153**, 521-534 (2013).
4. A. J. Pagán *et al.*, Myeloid growth factors promote resistance to mycobacterial infection by curtailing granuloma necrosis through macrophage replenishment. *Cell host & microbe* **18**, 15-26 (2015).
5. F. J. Roca, L. J. Whitworth, S. Redmond, A. A. Jones, L. Ramakrishnan, TNF induces pathogenic programmed macrophage necrosis in tuberculosis through a mitochondrial-lysosomal-endoplasmic reticulum circuit. *Cell* **178**, 1344-1361. e1311 (2019).
6. R. S. Wu *et al.*, A rapid method for directed gene knockout for screening in G0 zebrafish. *Developmental cell* **46**, 112-125. e114 (2018).
7. S. Garritano *et al.*, Determining the effectiveness of High Resolution Melting analysis for SNP genotyping and mutation scanning at the TP53 locus. *BMC genetics* **10**, 5 (2009).
8. K. Takaki, J. M. Davis, K. Winglee, L. Ramakrishnan, Evaluation of the pathogenesis and treatment of Mycobacterium marinum infection in zebrafish. *Nature protocols* **8**, 1114 (2013).
9. A. K. Singh, J. M. Reyrat, Laboratory maintenance of Mycobacterium smegmatis. *Current protocols in microbiology* **14**, 10C. 11.11-10C. 11.12 (2009).
10. S. L. Sampson *et al.*, Extended safety and efficacy studies of a live attenuated double leucine and pantothenate auxotroph of Mycobacterium tuberculosis as a vaccine candidate. *Vaccine* **29**, 4839-4847 (2011).
11. M. P. Weinstein, National Committee for Clinical Laboratory Standard, Methods for Broth and Agar Dilution Antimicrobial Susceptibility Testing Process; Approved Guideline, NCCLS document *M07-11th*, 15-84 (2018).
12. P. Willanova, National Committee for Clinical Laboratory Standard, Methods for Determining Bactericidal Activity of Antimicrobial Agents; Approved Guideline, NCCLS document. *M26-A* **19**, 18 (1999).
13. C. Barton (2018) Raising Larvae in the Zebrafish International Resource Center Autonursery. (ZFIN, https://zfin.atlassian.net/wiki/spaces/prot/pages/352157867/Raising+Larvae+in+the+Zebrafish+International+Resource+Center+Autonursery).
14. S. Levitte *et al.*, Mycobacterial acid tolerance enables phagolysosomal survival and establishment of tuberculous infection in vivo. *Cell host & microbe* **20**, 250-258 (2016).
15. L. T. Lelieveld *et al.*, Role of β-glucosidase 2 in aberrant glycosphingolipid metabolism: model of glucocerebrosidase deficiency in zebrafish. *J Lipid Res* **60**, 1851-1867 (2019).
16. L. T. Lelieveld *et al.*, Consequences of excessive glucosylsphingosine in glucocerebrosidase-deficient zebrafish. *Journal of Lipid Research* (2022).
17. Q. Su *et al.*, Xylose-Configured Cyclophellitols as Selective Inhibitors for Glucocerebrosidase. *Chembiochem* **22**, 3090-3098 (2021).
18. R. D. Berg *et al.*, Lysosomal Disorders Drive Susceptibility to Tuberculosis by Compromising Macrophage Migration. *Cell* **165**, 139-152 (2016).

19. E. Aflaki *et al.*, Macrophage models of Gaucher disease for evaluating disease pathogenesis and candidate drugs. *Science translational medicine* **6**, 240ra273-240ra273 (2014).
20. N. Dekker *et al.*, Elevated plasma glucosylsphingosine in Gaucher disease: relation to phenotype, storage cell markers, and therapeutic response. *Blood* **118**, e118-127 (2011).
21. M. Cuffe, Lodish, Harvey F. , Staehelin, L. Andrew , Stein, Wilfred D. , Chow, Christopher , Alberts, Bruce M. , Cooper, John A. , Laskey, Ronald A. , Bernfield, Merton R. and Slack, Jonathan M.W.. (2021) The structure of biological molecules. in *Cell Biology-The nature and function of cells* (Encyclopedia Britannica).
22. R. Milo, R. Philips, *Cell Biology by the Numbers: What is the Density of Cells? Bionumbers* (Garland Science, New York, ed. 1st, 2015), https://doi.org/10.1201/9780429258770.
23. G. Cooper, *The Cell: A Molecular Approach. 2nd edition*, The Molecular Composition of Cells (2000).
